# Supplementary material for: PEAC-seq adopts Prime Editor to detect CRISPR off-target and DNA translocation
Source: Nat Commun. 2022 Dec 12;13:7545. doi: 10.1038/s41467-022-35086-8 (PMC9744820; doi:10.1038/s41467-022-35086-8)
Supplement: Supplementary file 1 — Supplementary Information [file 41467_2022_35086_MOESM1_ESM.pdf]

Supplementary Fig. 1

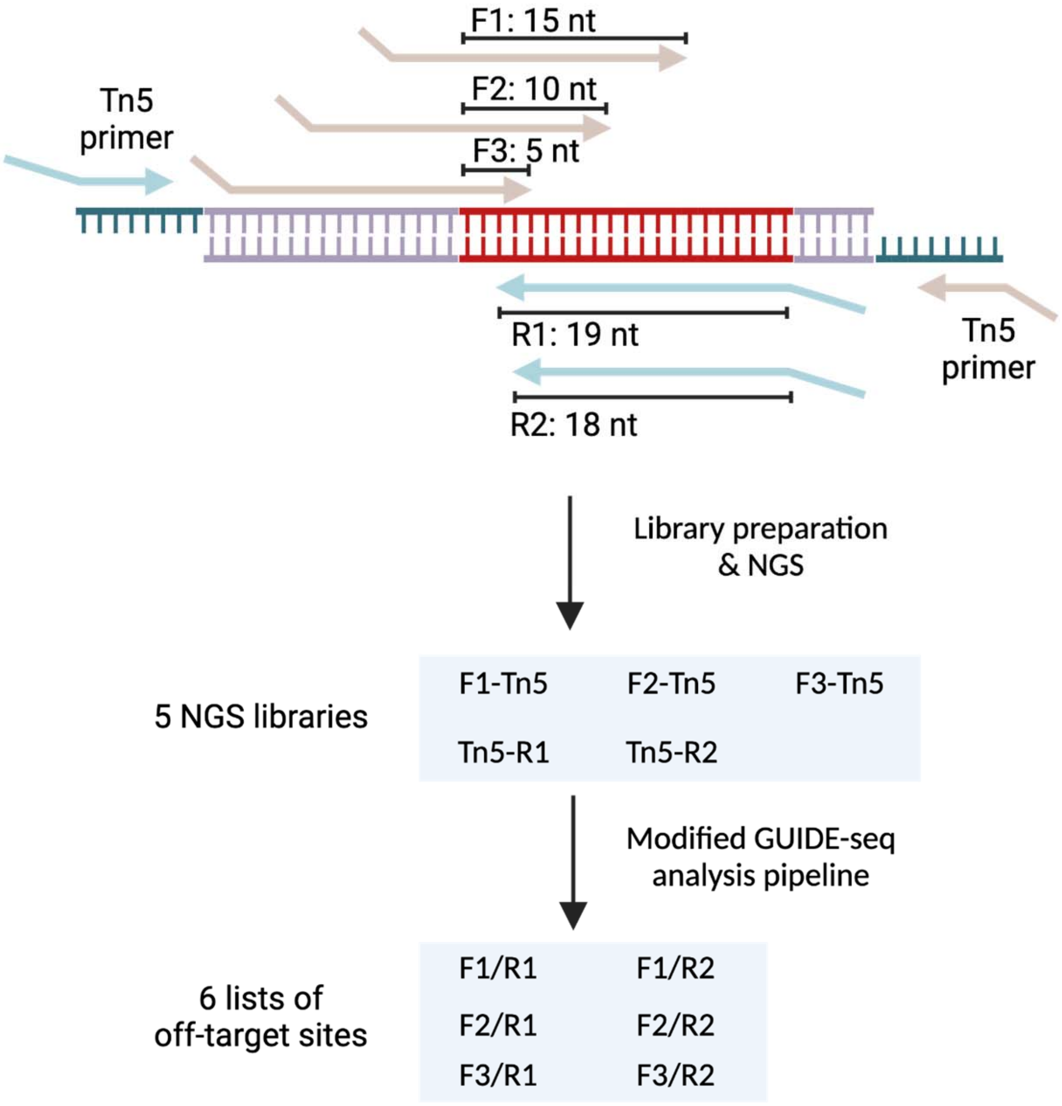

sFig. 1 | Library preparation and modified GUIDE-seq pipeline to generate six lists of candidate sites
Amplicons were enriched by PEAC-seq insertion-specific primers and Tn5 primers. Three forward primers and two reverse primers were used with the upstream (light blue) and downstream (yellow) Tn5 primers, in five separate PCR reactions. A total of five NGS libraries were generated and sequenced. A modified GUIDE-seq analysis pipeline was applied and generated six lists of candidate sites from each pair of the forward and the reverse primers.

Supplementary Fig. 2

a

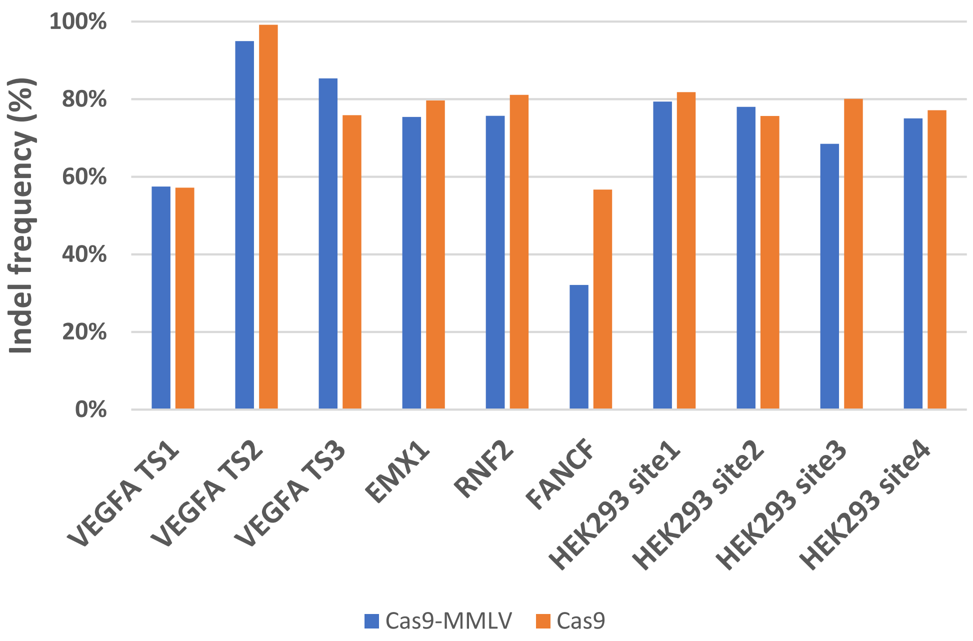

b

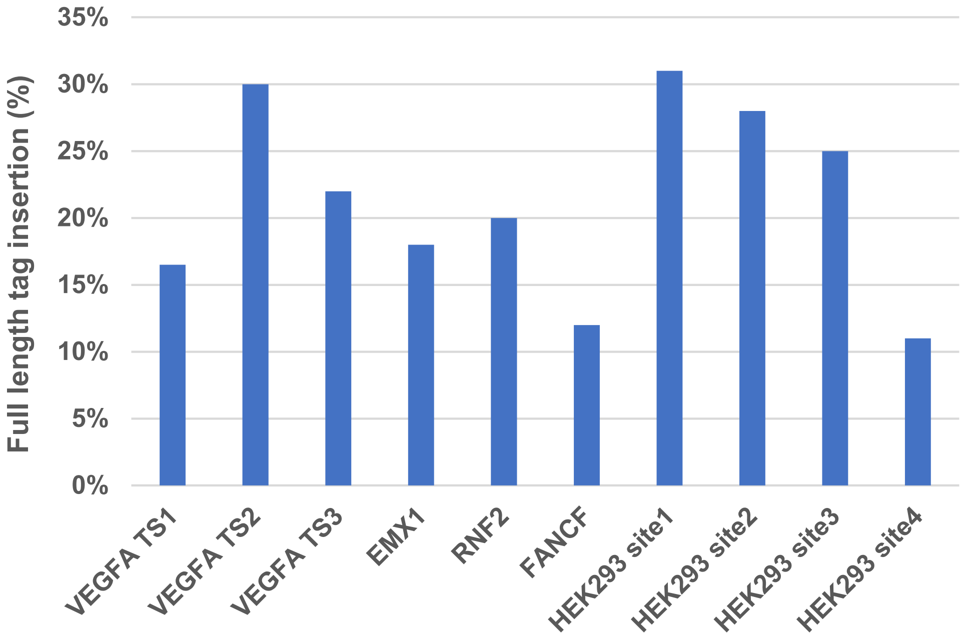

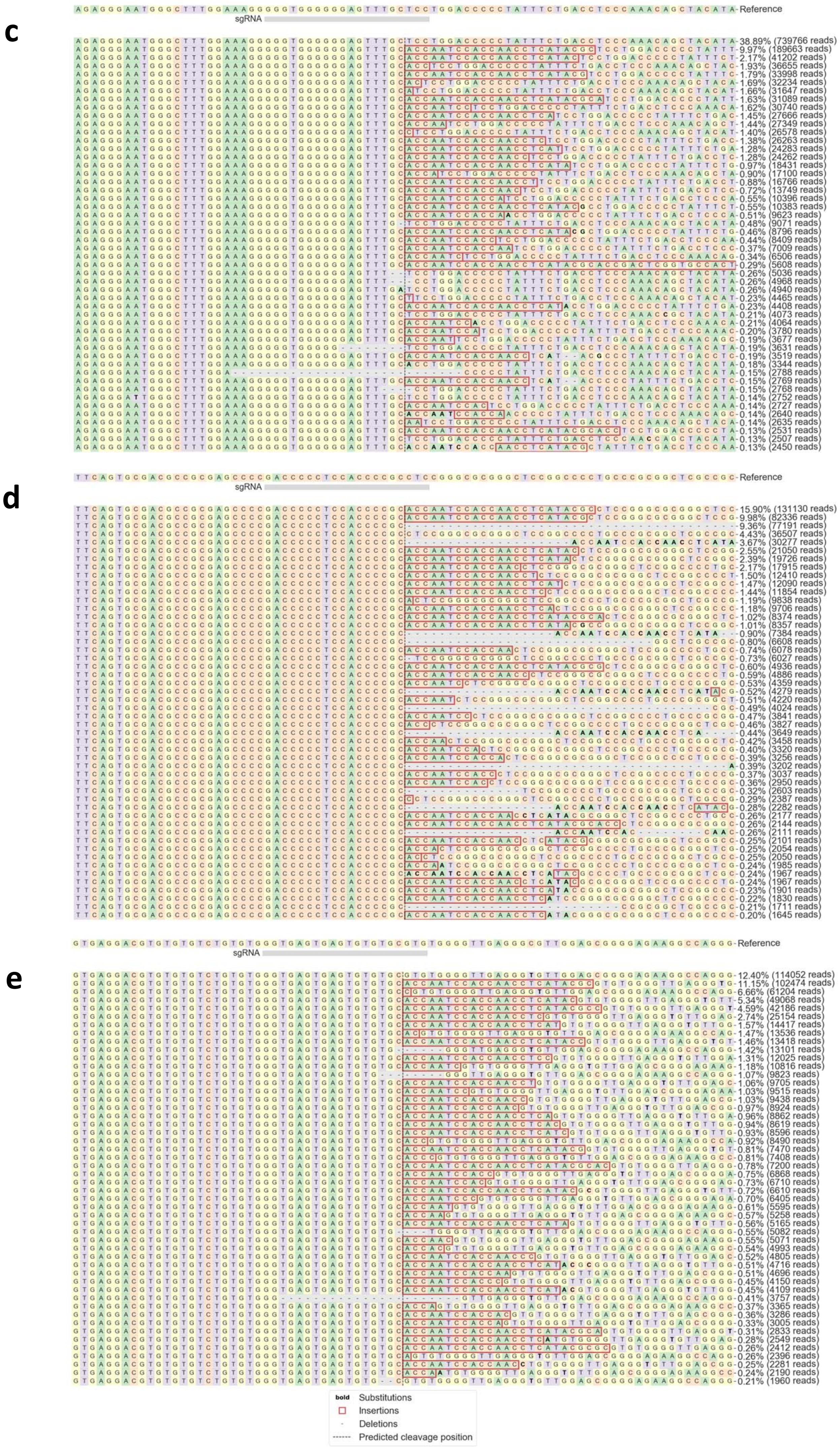

c
Reference
sgRNA
d
Reference
sgRNA
e
Reference
sgRNA
bold Substitutions
Insertions
Deletions
Predicted cleavage position

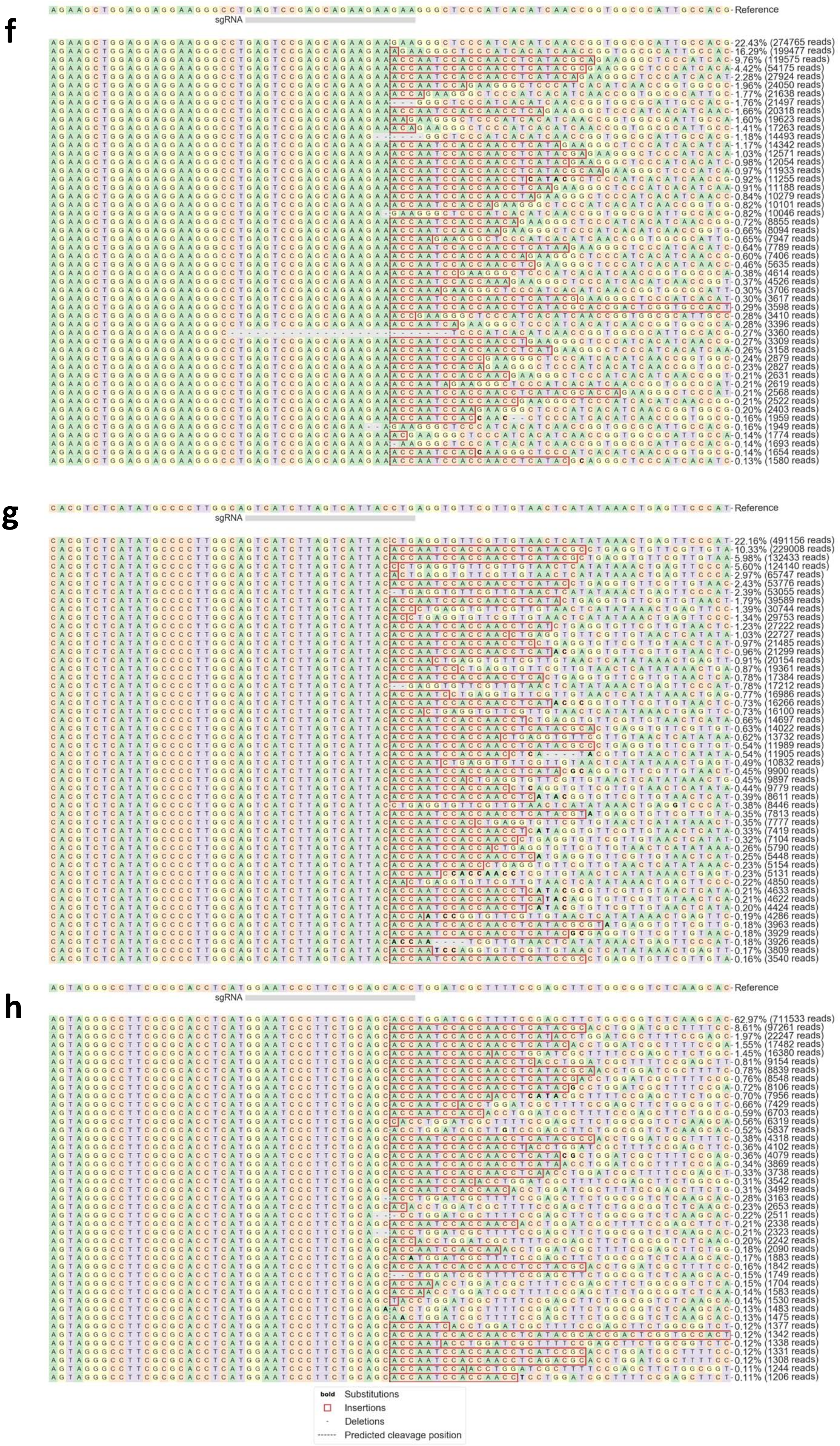

f
g
h
Reference
sgRNA
bold Substitutions
Insertions
Deletions
Predicted cleavage position

sFig. 2 | The indel frequency and tag insertion ability of the Cas9-MMLV system.

a. We conducted Amplicon-seq to quantify the indel frequency of ten on-target sites. The indels were generated by Cas9 or Cas9-MMLV. **b.** The frequency of tag insertion was estimated on the same ten on-target sites. Source data are provided as a Source Data file. c-h. The PEAC-seq tag insert efficiency at *VEGFA TS1* **(c)**, *VEGFA TS2* **(d)**, *VEGFA TS3* **(e)**, *EMX1* **(f)**, *RNF2* **(g)**, and *FANCF* **(h)** analyzed by CRISPResso2.

Supplementary Fig. 3

PEAC-ID	Target location (hg38)	VEGFA TS1 IGV Screenshot	Compare to GUIDE-seq
PEAC-1	chr6:43769553-43769576	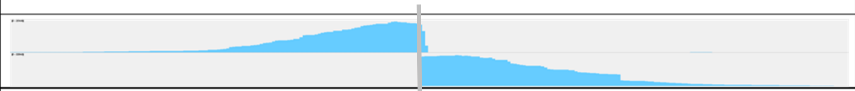	Overlap
PEAC-2	chr15:65345192-65345215	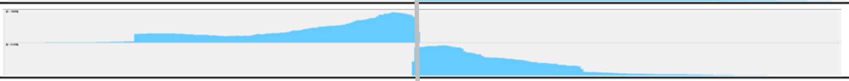	Overlap
PEAC-3	chr12:131205636-131205659	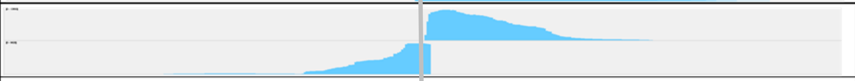	Overlap
PEAC-4	chr1:98882088-98882111	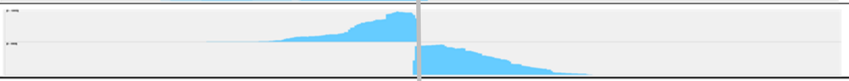	Overlap
PEAC-5	chr12:1878893-1878916	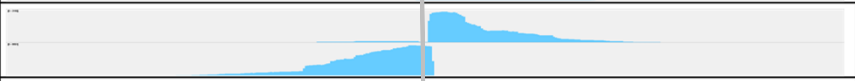	Overlap
PEAC-6	chr17:41640069-41640092	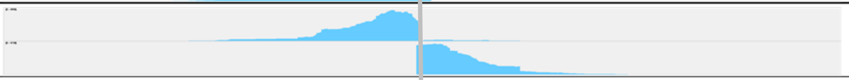	Overlap
PEAC-7	chr6:14316135-14316158	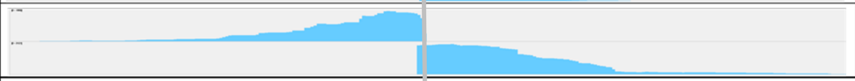	Overlap
PEAC-8	chr22:36819214-36819237	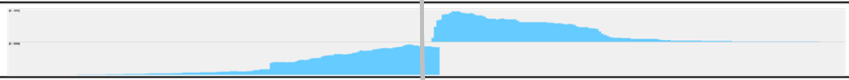	Overlap
PEAC-9	chr17:49240170-49240193	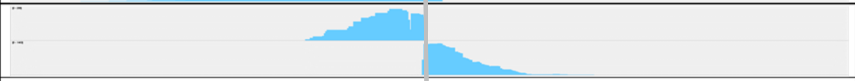	Overlap
PEAC-10	chr5:32945151-32945174	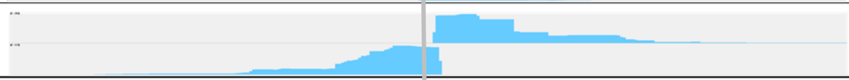	Overlap
PEAC-11	chr22:19710933-19710956	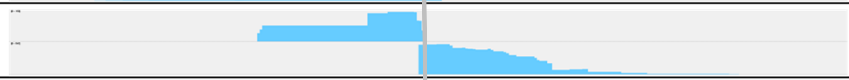	Overlap
PEAC-12	chr5:56876245-56876268	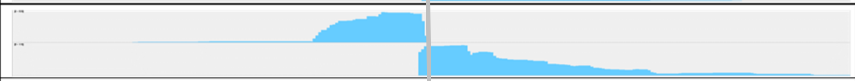	Overlap
PEAC-13	chr4:8452058-8452081	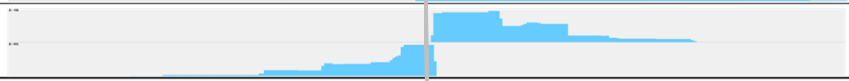	Overlap
PEAC-14	chr1:233021590-233021613	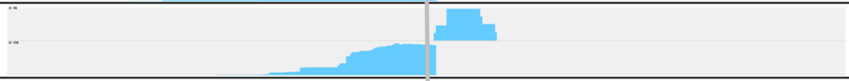	Overlap
PEAC-15	chr1:33177669-33177692	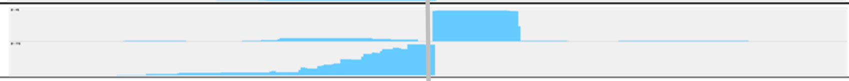	PEAC-seq Unique
PEAC-16	chr10:122971882-122971905	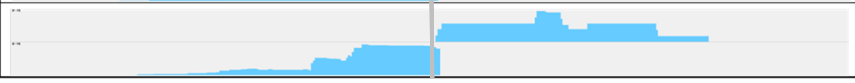	PEAC-seq Unique

Cut site

sFig. 3 | Signal tracks of PEAC-seq at VEGFA TS1

The signal tracks of PEAC-seq at VEGFA TS1 were shown here. Chromosome locations and the overlap with GUIDE-seq were also shown.

Supplementary Fig. 4a

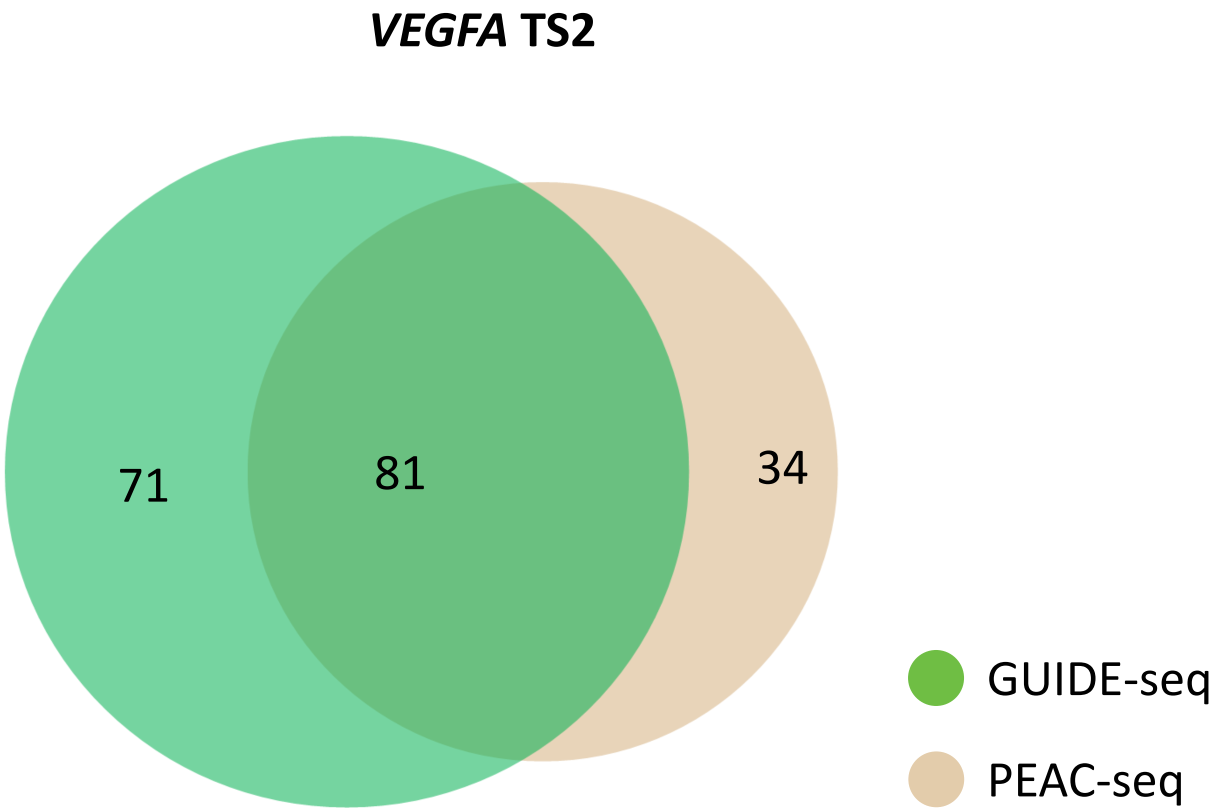

Supplementary Fig. 4b

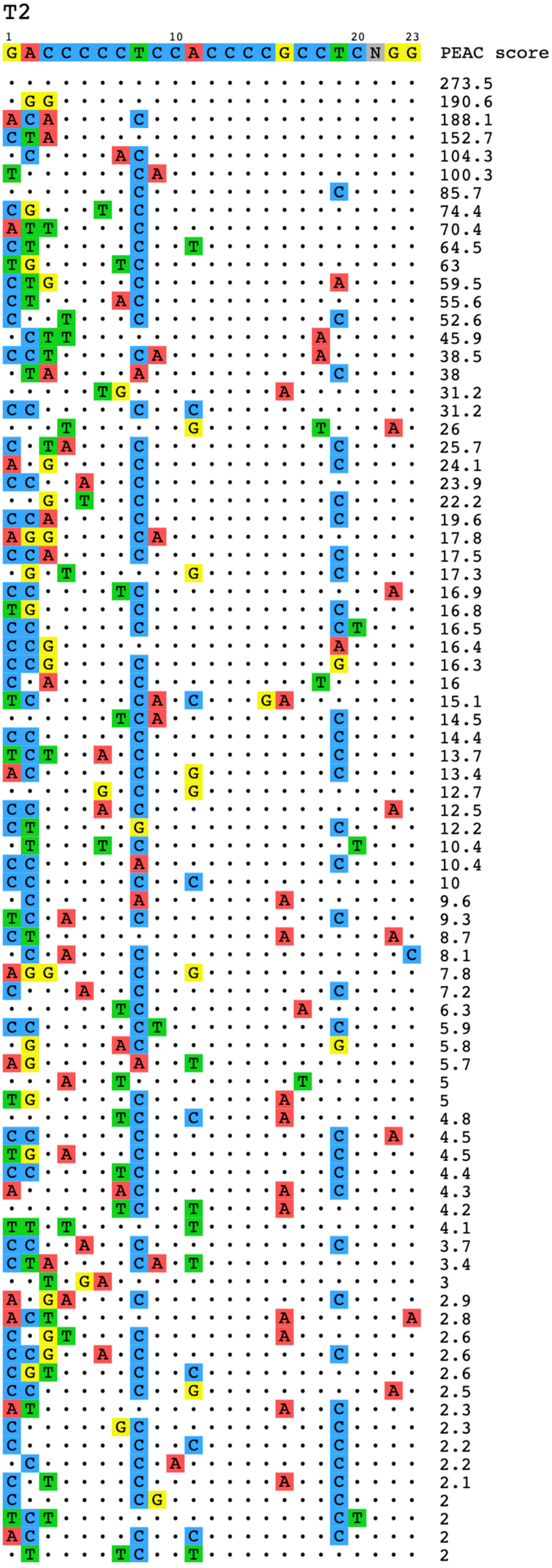

Supplementary Fig. 4c

Target location (hg38)	VEGFA TS2 IGV Screenshot	Compare to GUIDE-seq
chr6:43770818-43770841	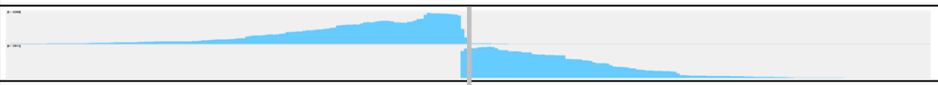	overlap
chr11:31795928-31795951	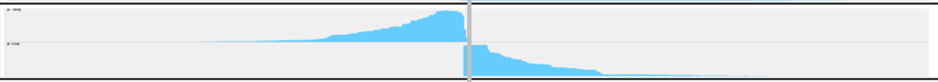	overlap
chr9:100837360-100837383	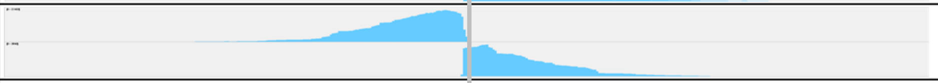	overlap
chr5:6714988-6715011	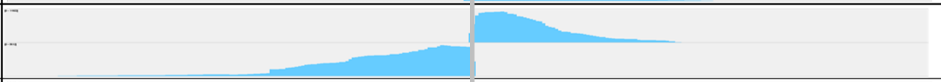	overlap
chr18:23779588-23779611	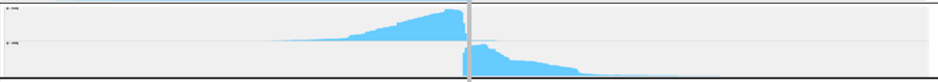	overlap
chr17:4455450-4455473	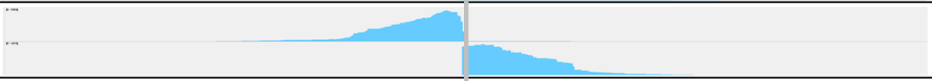	overlap
chr15:32993899-32993922	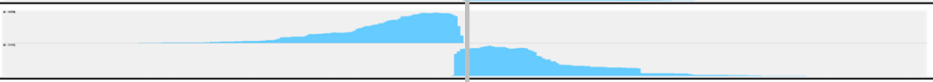	overlap
chr10:133336426-133336449	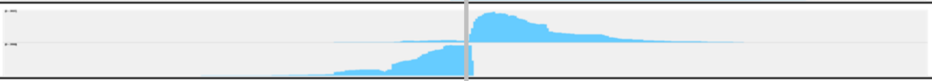	overlap
chr2:241275174-241275197	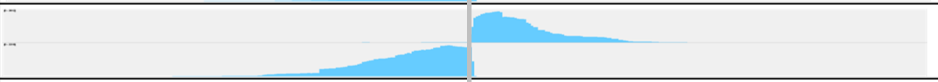	overlap
chr5:139648654-139648677	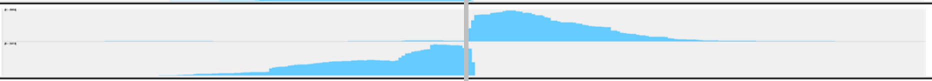	overlap
chr17:41888497-41888520	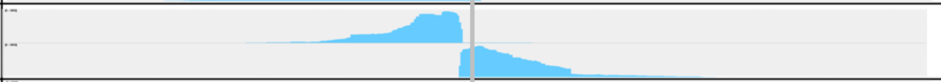	overlap
chrX:150764038-150764061	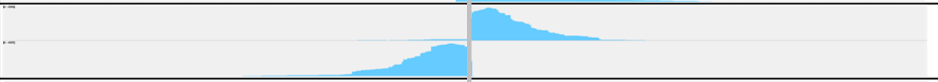	overlap
chr4:38535989-38536012	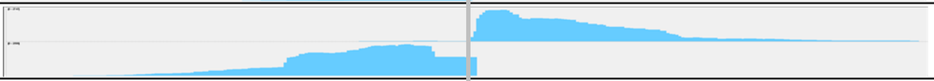	overlap
chr9:123375895-123375918	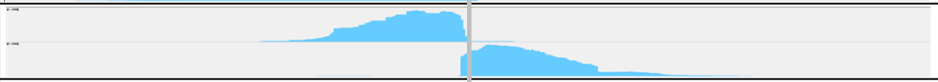	overlap
chr11:72237743-72237766	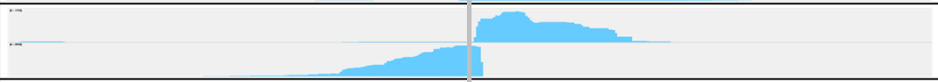	overlap
chr1:151059392-151059415	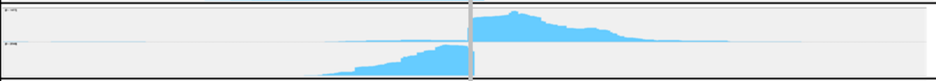	overlap
chr8:143740774-143740797	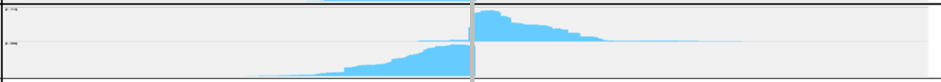	overlap
chr1:939940-939963	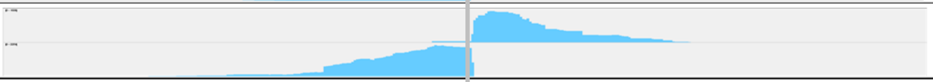	overlap
chr22:50446356-50446379	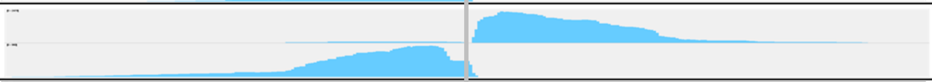	overlap
chr5:180161284-180161307	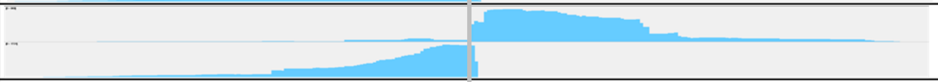	PEACSeq unique
chr4:8840184-8840207	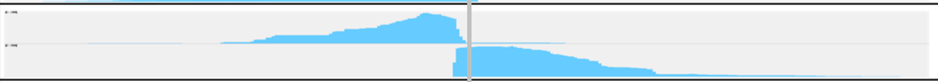	overlap
chr9:137368986-137369009	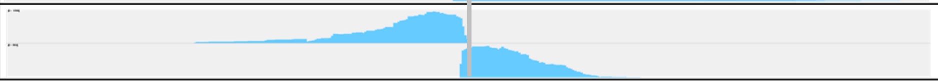	overlap

Cut site

Supplementary Fig. 4c (continued)

Target location (hg38)	VEGFA TS2 IGV Screenshot	Compare to GUIDE-seq
chr6:43770818-43770841	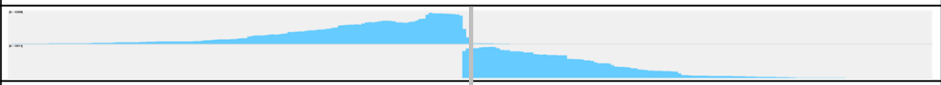	overlap
chr11:31795928-31795951	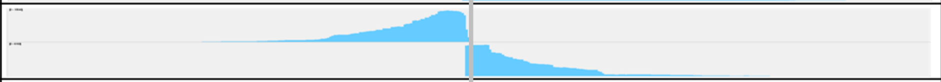	overlap
chr9:100837360-100837383	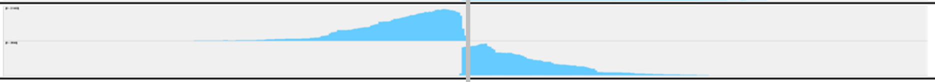	overlap
chr5:6714988-6715011	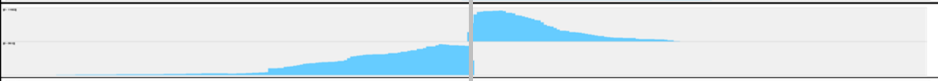	overlap
chr18:23779588-23779611	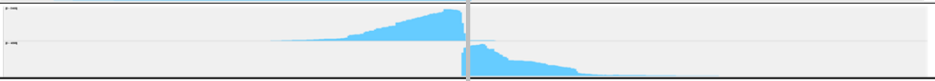	overlap
chr17:4455450-4455473	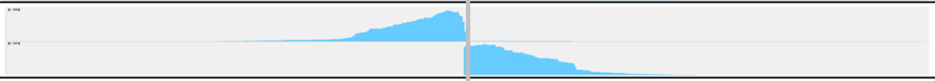	overlap
chr15:32993899-32993922	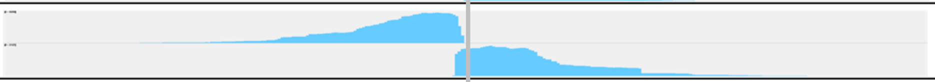	overlap
chr10:133336426-133336449	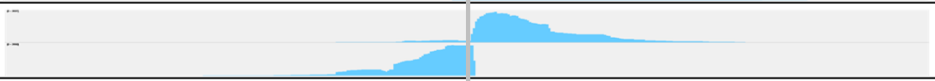	overlap
chr2:241275174-241275197	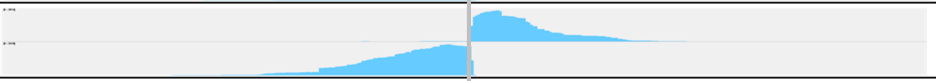	overlap
chr5:139648654-139648677	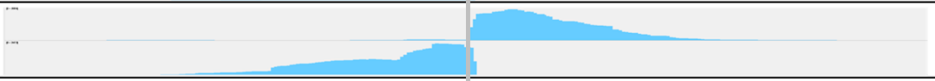	overlap
chr17:41888497-41888520	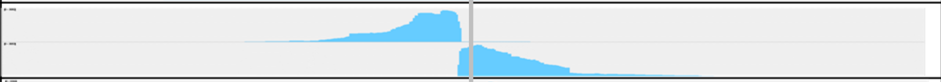	overlap
chrX:150764038-150764061	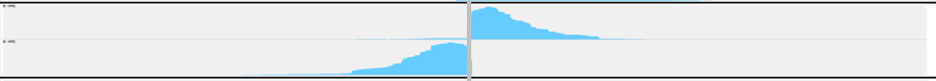	overlap
chr4:38535989-38536012	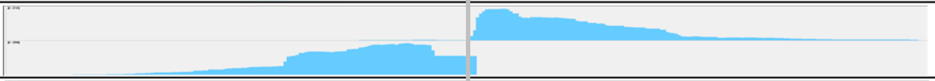	overlap
chr9:123375895-123375918	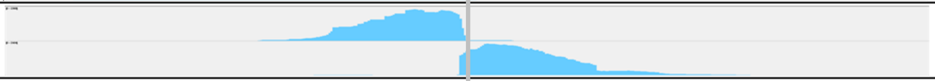	overlap
chr11:72237743-72237766	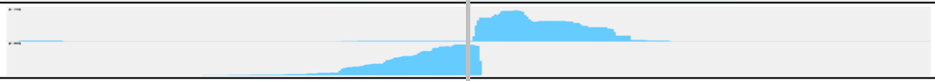	overlap
chr1:151059392-151059415	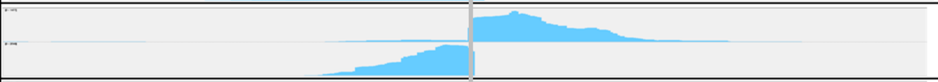	overlap
chr8:143740774-143740797	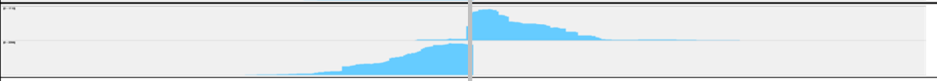	overlap
chr1:939940-939963	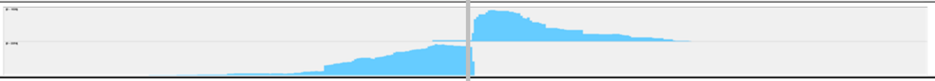	overlap
chr22:50446356-50446379	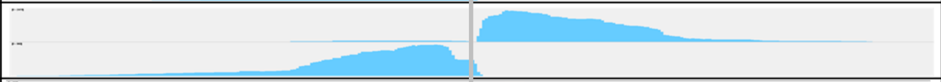	overlap
chr5:180161284-180161307	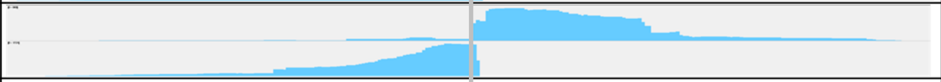	PEACSeq unique
chr4:8840184-8840207	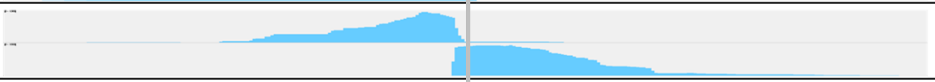	overlap
chr9:137368986-137369009	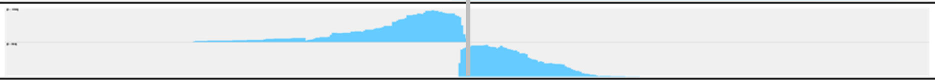	overlap

Cut site

Supplementary Fig. 4c (continued)

Target location (hg38)	VEGFA TS2 IGV Screenshot (continued)	Compare to GUIDE-seq
chr10:114534490-114534513	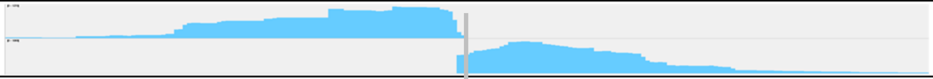	overlap
chr19:14032156-14032179	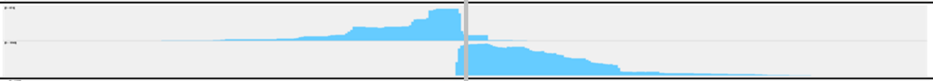	overlap
chr20:10933306-10933329	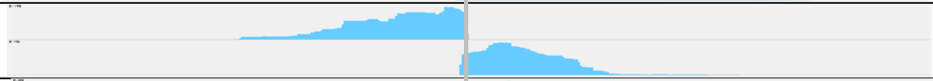	overlap
chr4:1494513-1494536	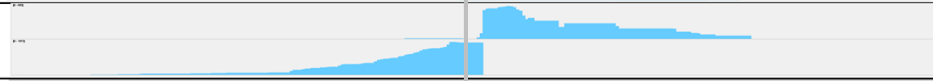	overlap
chr2:169716823-169716846	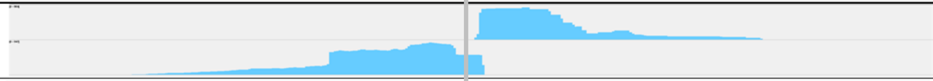	overlap
chr11:65712291-65712314	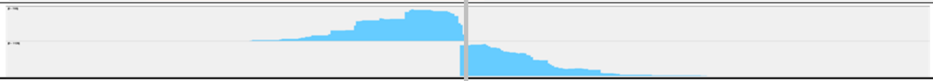	overlap
chr9:37465350-37465373	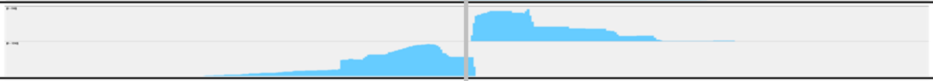	overlap
chrX:129906647-129906670	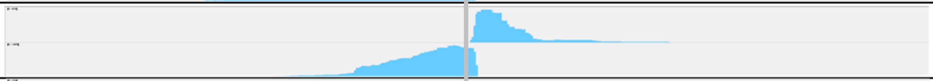	overlap
chr11:19777094-19777117	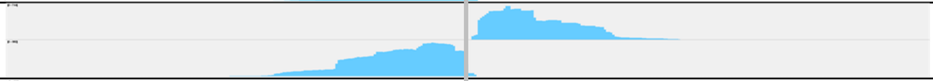	PEACSeq unique
chr17:57663161-57663184	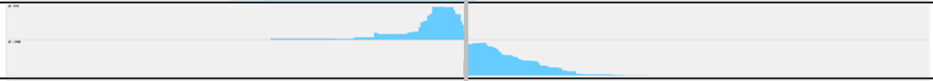	overlap
chr5:171451048-171451071	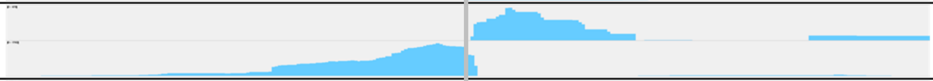	overlap
chr3:140679941-140679964	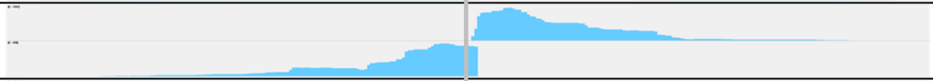	overlap
chr13:26052087-26052110	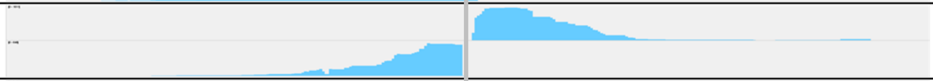	overlap
chr7:95690358-95690381	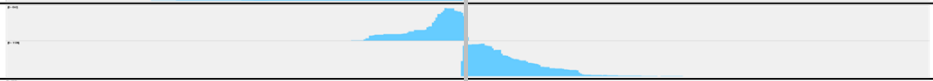	overlap
chr2:128486621-128486644	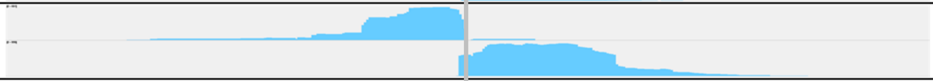	overlap
chr1:77779744-77779767	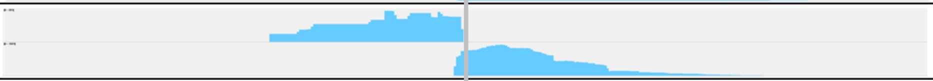	PEACSeq unique
chr11:73747435-73747458	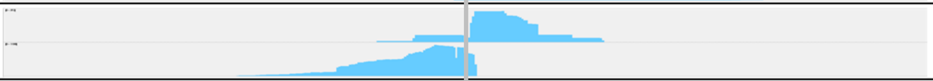	PEACSeq unique
chr1:11654471-11654494	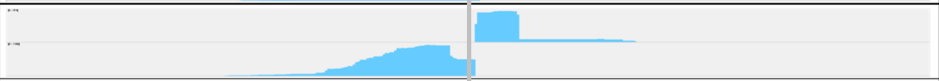	PEACSeq unique
chr9:127900790-127900813	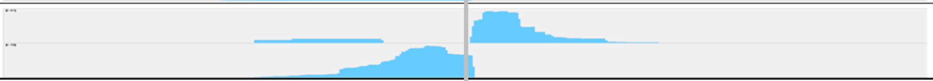	overlap
chr17:17051531-17051554	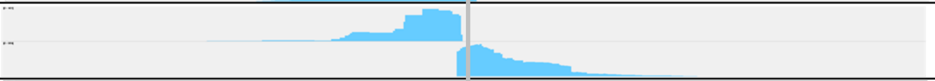	overlap
chr22:39706378-39706401	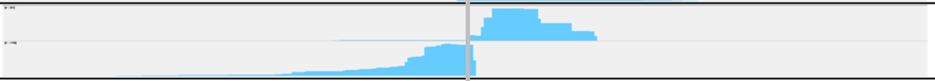	overlap
chr8:18184030-18184053	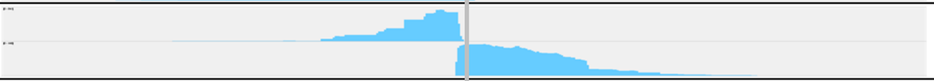	overlap
chr13:99894728-99894751	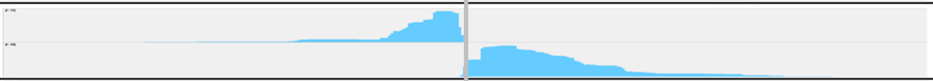	overlap
chr19:13011357-13011380	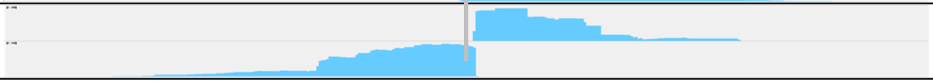	overlap

Cut site

Supplementary Fig. 4c (continued)

Target location (hg38)	VEGFA TS2 IGV Screenshot (continued)	Compare to GUIDE-seq
chr7:105293491-105293514	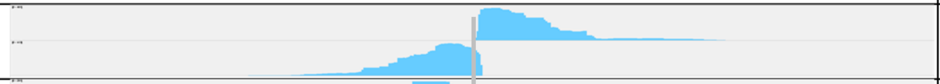	overlap
chr6:31495043-31495066	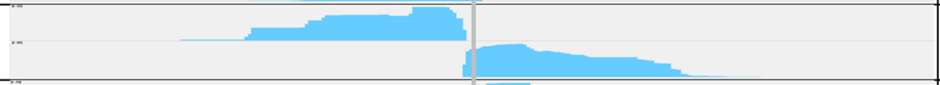	overlap
chr6:98825031-98825054	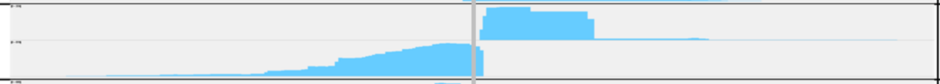	PEACSeq unique
chr11:374660-374683	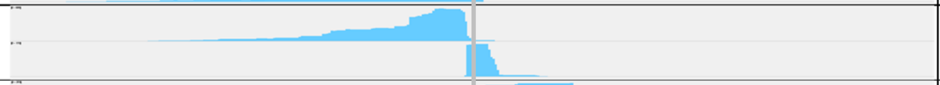	overlap
chr3:195762332-195762355	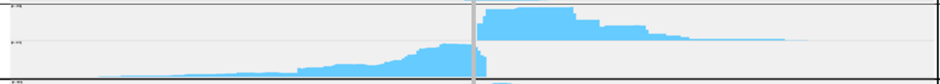	PEACSeq unique
chr9:27338859-27338882	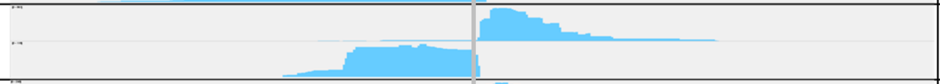	overlap
chr19:44821307-44821330	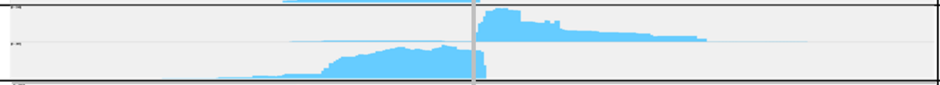	overlap
chr19:29845075-29845098	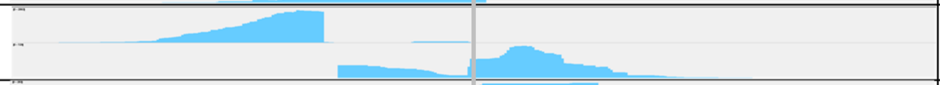	PEACSeq unique
chr22:43288472-43288495	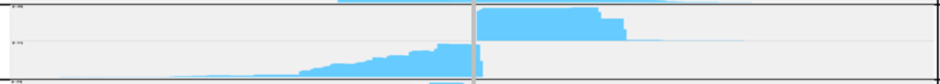	overlap
chr11:63623611-63623634	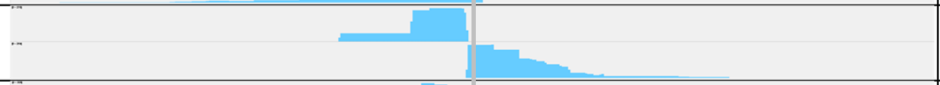	overlap
chr16:56929510-56929533	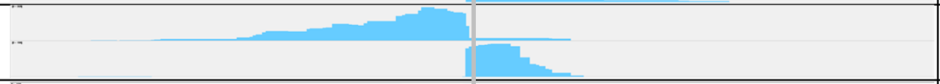	overlap
chr12:57087439-57087462	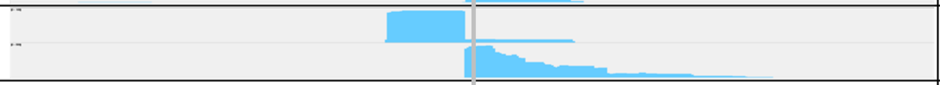	PEACSeq unique
chr12:22334709-22334732	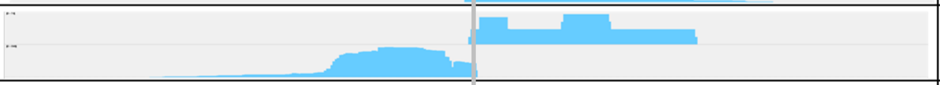	PEACSeq unique
chr5:133524667-133524690	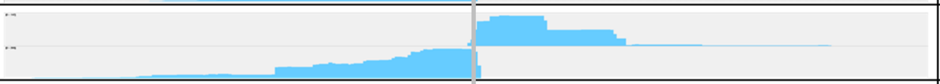	overlap
chr1:26696329-26696352	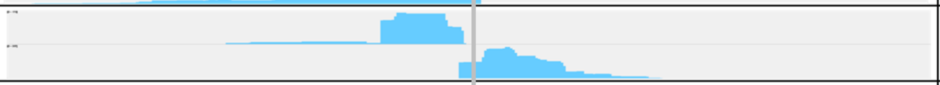	PEACSeq unique
chr8:103500163-103500186	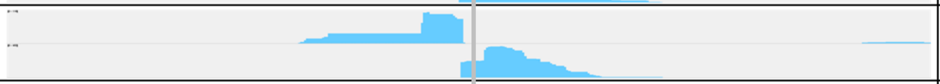	PEACSeq unique
chr1:50976513-50976536	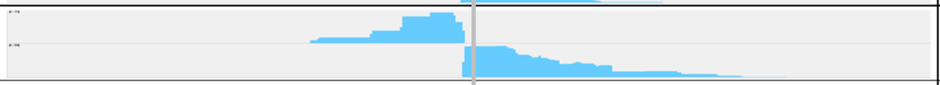	PEACSeq unique
chr19:42302642-42302665	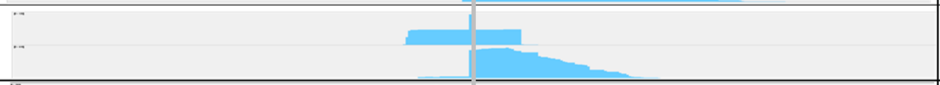	overlap
chr3:38099767-38099790	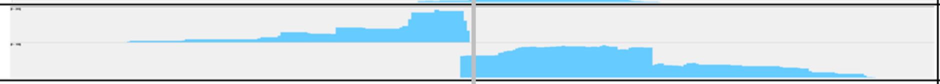	overlap
chr10:101061743-101061766	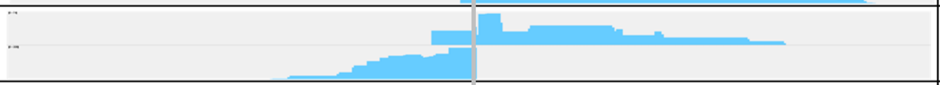	overlap
chr12:31659398-31659421	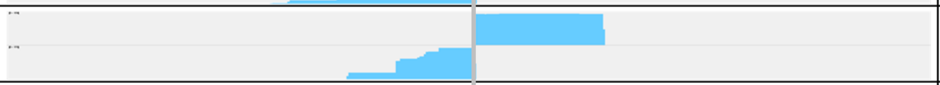	PEACSeq unique
chr19:45448779-45448802	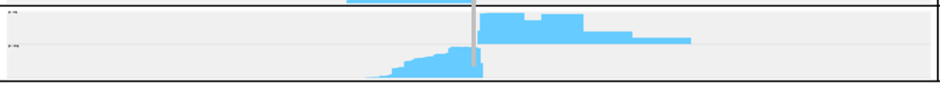	overlap

Cut site

Supplementary Fig. 4c (continued)

Target location (hg38)	VEGFA TS2 IGV Screenshot(continued)	Compare to GUIDE-seq
chr1:23435751-23435774	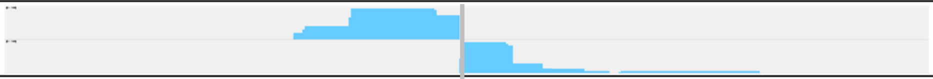	overlap
chr10:70778444-70778467	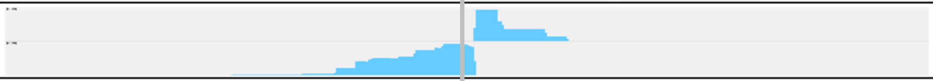	overlap
chr15:84748852-84748875	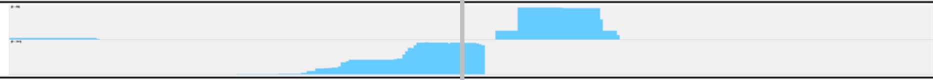	PEACSeq unique
chr21:45283300-45283323	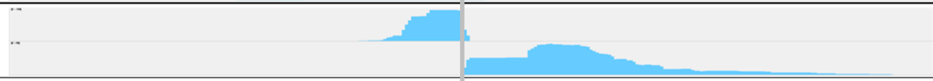	overlap
chr10:100969482-100969505	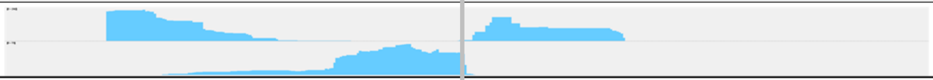	PEACSeq unique
chr11:12287370-12287393	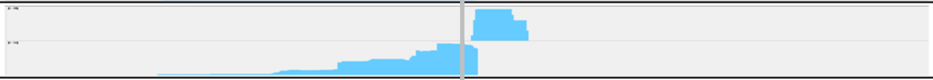	overlap
chr8:37393659-37393682	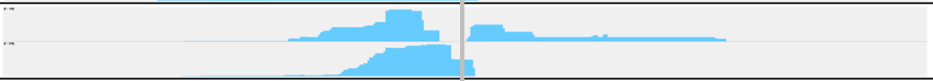	overlap
chr17:81923260-81923283	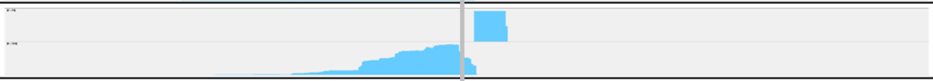	overlap
chr4:7327802-7327825	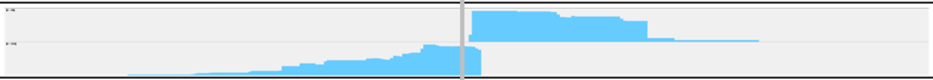	overlap
chr11:46120297-46120320	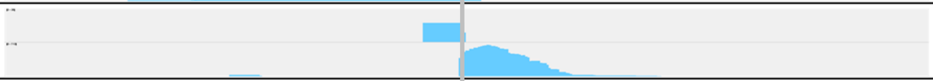	overlap
chr11:32162932-32162955	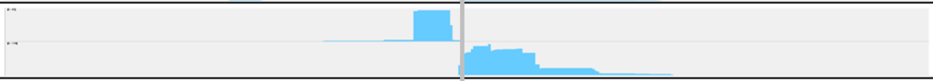	overlap
chr11:133907545-133907568	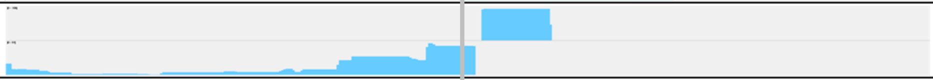	overlap
chr2:24634707-24634730	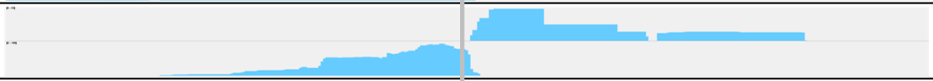	overlap
chr9:33202609-33202632	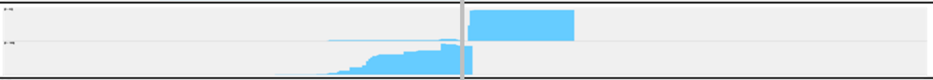	overlap
chr11:72750152-72750175	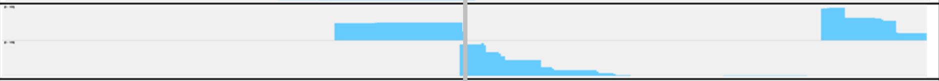	PEACSeq unique
chr2:47570989-47571012	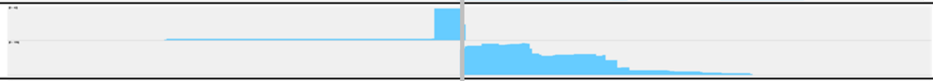	PEACSeq unique
chr7:50697814-50697837	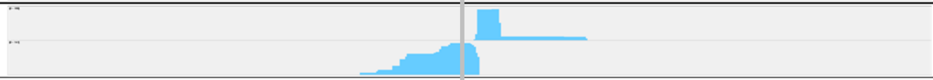	overlap
chr19:58154691-58154714	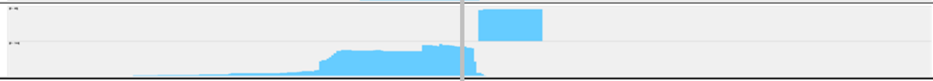	PEACSeq unique
chr11:13926806-13926829	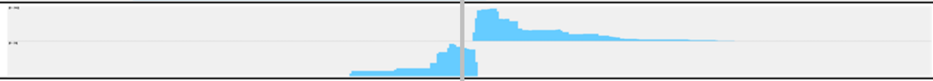	overlap
chr11:83722550-83722573	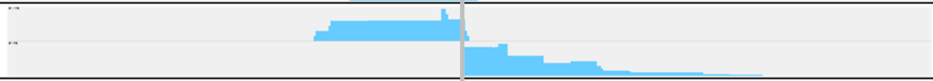	overlap
chr3:31417159-31417182	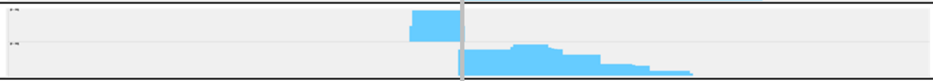	overlap
chr19:18020497-18020520	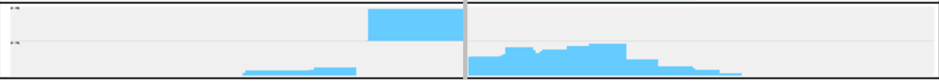	overlap

Cut site

Supplementary Fig. 4c (continued)

Target location (hg38)	VEGFA TS2 IGV Screenshot (continued)	Compare to GUIDE-seq
chr19:51412258-51412281		overlap
chr9:133572178-133572201		PEACSeq unique
chr13:51767773-51767796		PEACSeq unique
chr14:104134854-104134877		overlap
chr2:222559160-222559183		PEACSeq unique
chr3:13145795-13145818		overlap
chr2:169967228-169967251		PEACSeq unique
chr4:142846213-142846236		overlap
chr11:61553960-61553983		overlap
chr20:63944457-63944480		overlap
chr6:2737969-2737992		PEACSeq unique
chr1:27235142-27235165		PEACSeq unique
chr12:68807995-68808018		PEACSeq unique
chr17:48026474-48026497		overlap
chr6:110199814-110199837		overlap
chr16:8403326-8403349		PEACSeq unique
chr17:64089687-64089710		overlap
chr2:66556906-66556929		PEACSeq unique
chr14:89936960-89936983		overlap
chr8:67343376-67343399		PEACSeq unique
chr15:74690775-74690798		PEACSeq unique
chr2:239424835-239424858		PEACSeq unique
chr1:205749914-205749937		PEACSeq unique
chr14:25322642-25322665		PEACSeq unique
chr20:2321725-2321748		PEACSeq unique

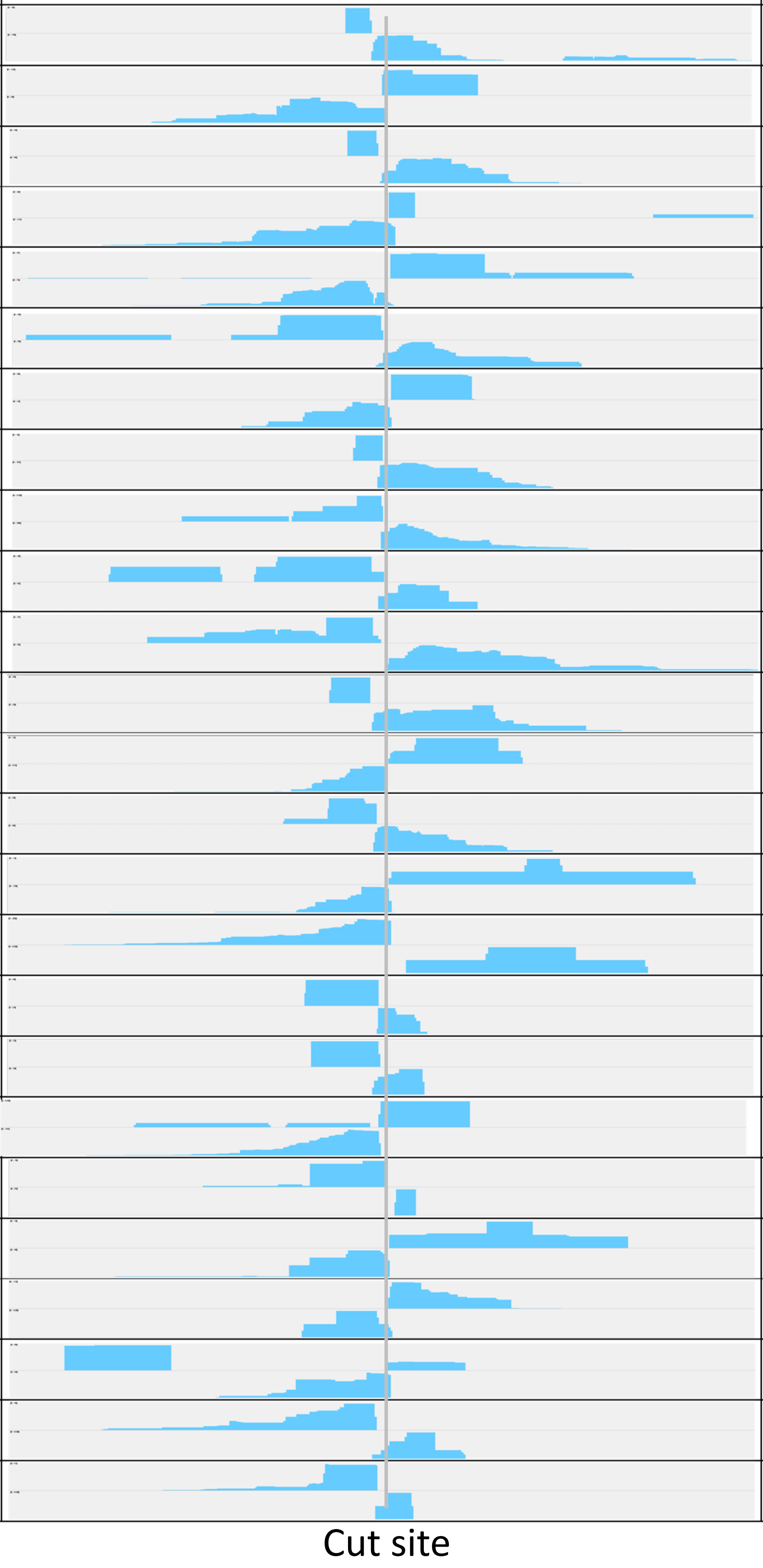

sFig. 4 | Signal tracks of PEAC-seq at VEGFA TS2

a. The Venn diagram shows the overlap of on-target and off-targets of VEGFA TS2 between the PEAC-seq and GUIDE-seq. Eighty-one sites were overlapped. Seventy-one sites were GUIDE-seq unique and thirty-four sites were PEAC-seq unique.

b. The GUIDE-seq visualization output of PEAC-seq sites at VEGFA TS2.

c. The signal tracks of PEAC-seq sites at VEGFA TS2. Chromosome locations and the overlap with GUIDE-seq were also shown. Source data are provided as a Source Data file.

Supplementary Fig. 5

a

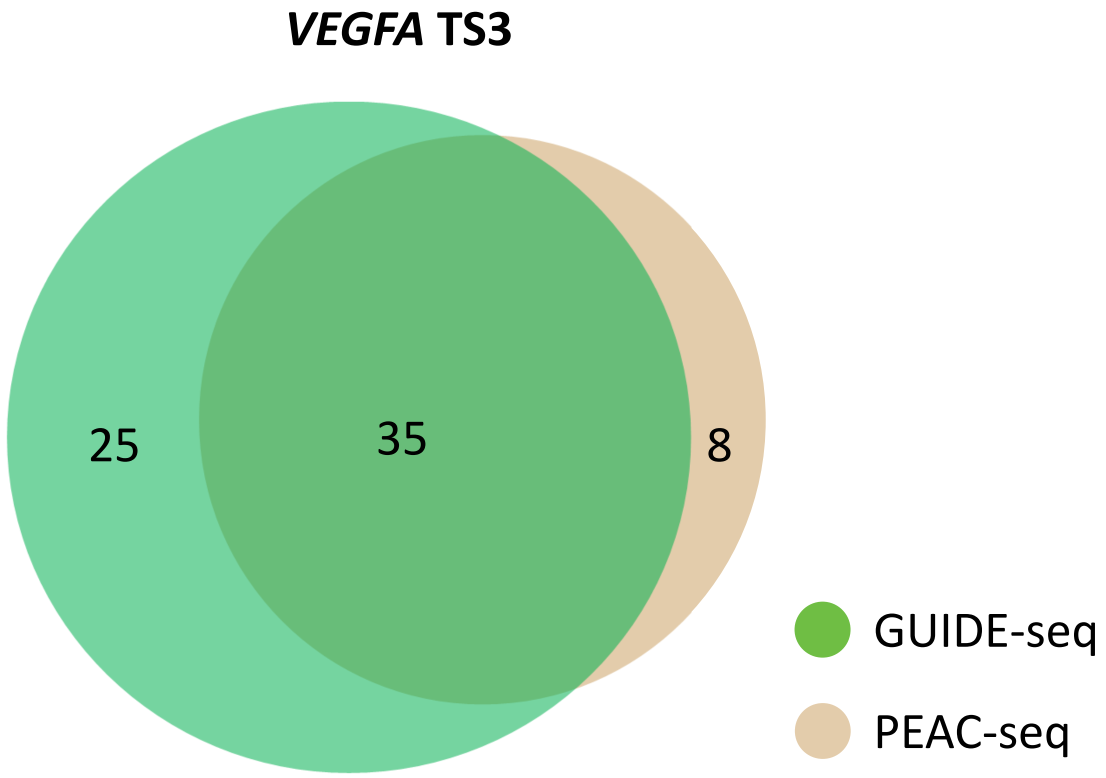

b

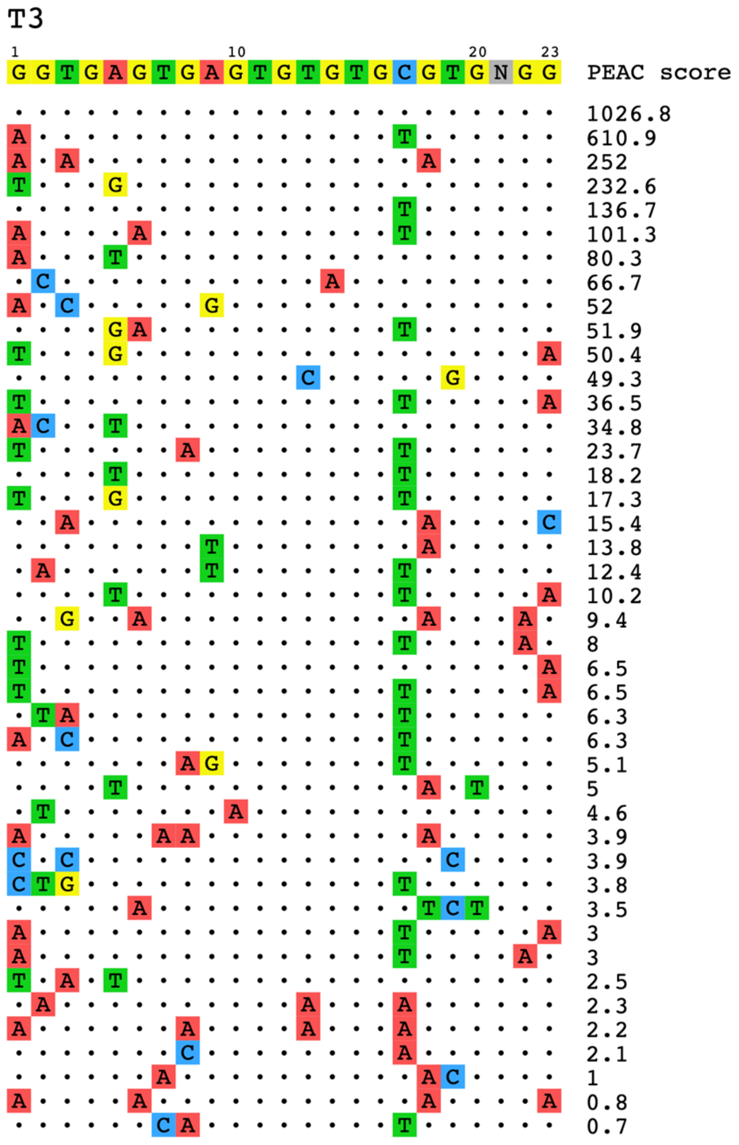

Supplementary Fig. 5

c

Target location (hg38)	VEGFA TS3 IGV Screenshot	Compare to GUIDE-seq
chr6:43769716-43769739	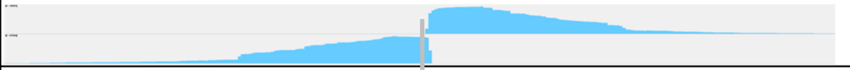	Overlap
chr14:65102434-65102457	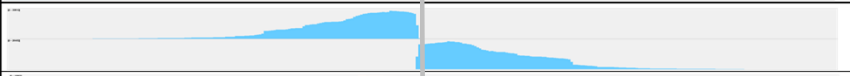	Overlap
chr5:90145145-90145168	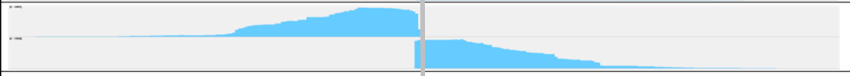	Overlap
chr5:116098961-116098984	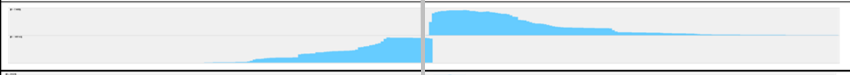	Overlap
chr14:105562677-105562700	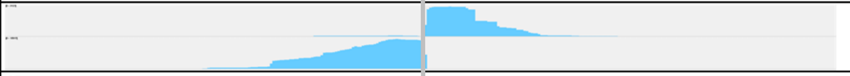	Overlap
chr3:194276088-194276111	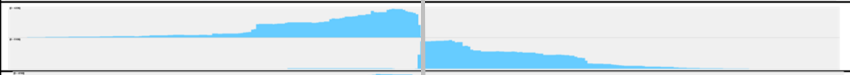	Overlap
chr20:20197633-20197656	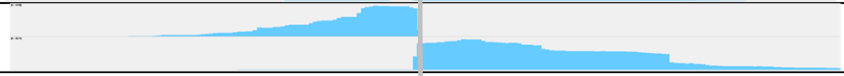	Overlap
chr22:37266776-37266799	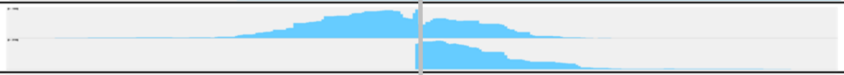	Overlap
chr14:73886776-73886799	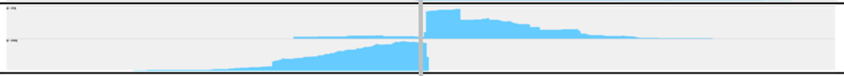	Overlap
chr12:6827873-6827896	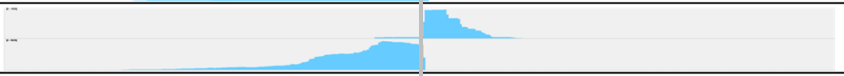	Overlap
chr9:23824549-23824572	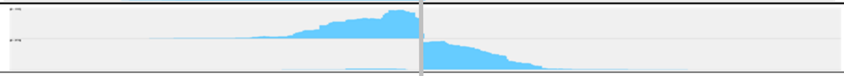	Overlap
chr11:69083657-69083680	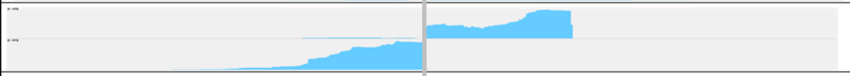	Overlap
chr19:6109004-6109027	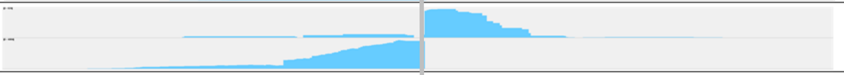	Overlap
chr19:40055953-40055976	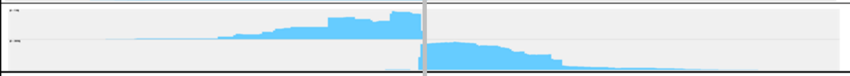	Overlap
chr14:61612048-61612071	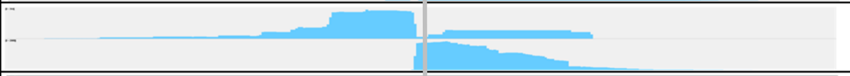	Overlap
chr22:49344058-49344081	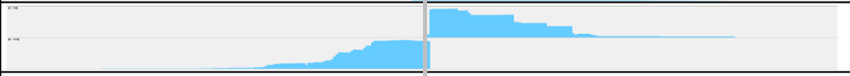	Overlap
chr1:47839359-47839382	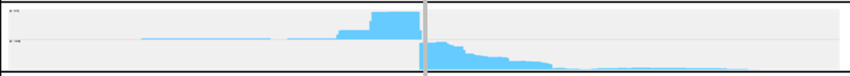	Overlap
chr1:181588050-181588073	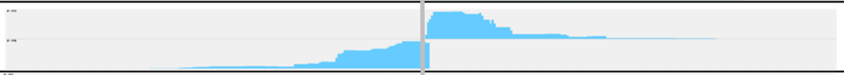	Overlap
chr2:176598691-176598714	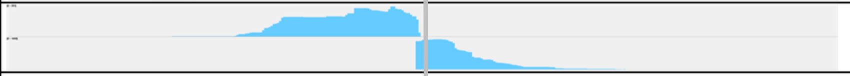	Overlap
chr4:61201894-61201917	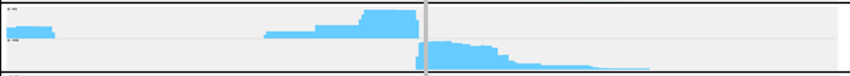	Overlap
chr7:39301509-39301532	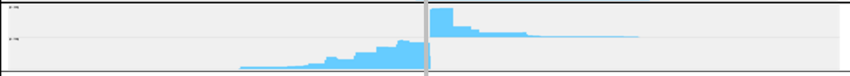	PEACSeq Unique
chr1:212466418-212466441	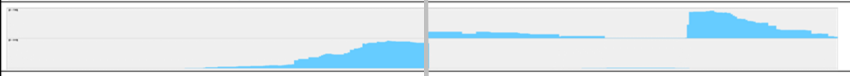	PEACSeq Unique
chr19:16458669-16458692	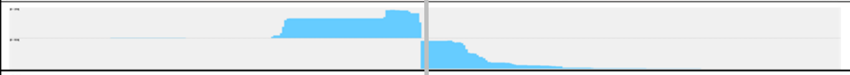	PEACSeq Unique
chr16:79982416-79982439	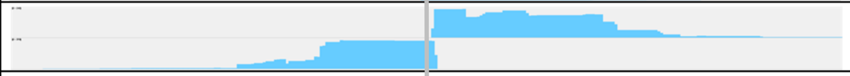	Overlap
chr8:23074983-23075006	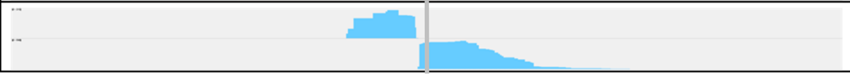	Overlap

Cut site

Supplementary Fig. 5 (continued)

Target location (hg38)	VEGFA TS3 IGV Screenshot	Compare to GUIDE-seq
chr8:48085228-48085251	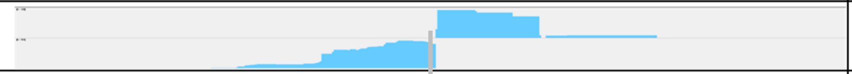	Overlap
chr9:18733630-18733653	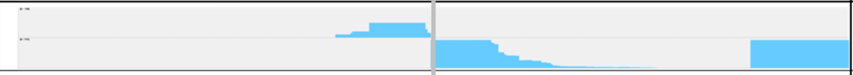	Overlap
chr11:7604557-7604580	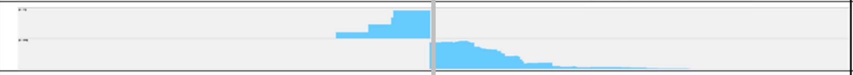	PEACSeq Unique
chr6:39060848-39060871	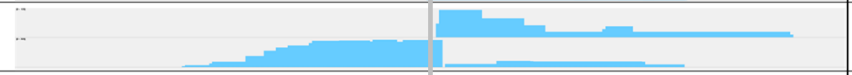	PEACSeq Unique
chr10:97000824-97000847	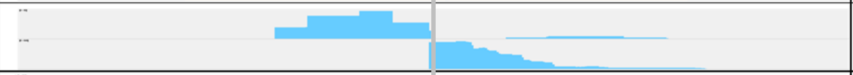	Overlap
chr2:18514941-18514964	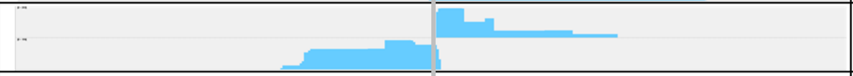	Overlap
chr3:71583651-71583674	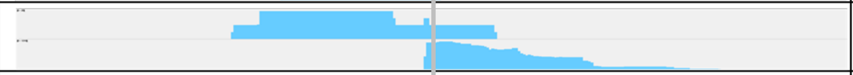	Overlap
chr19:47229228-47229251	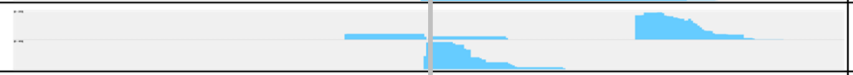	Overlap
chr16:83999034-83999057	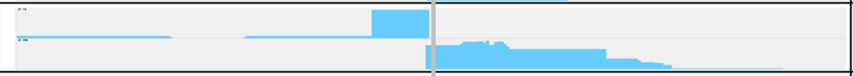	Overlap
chr16:12170738-12170761	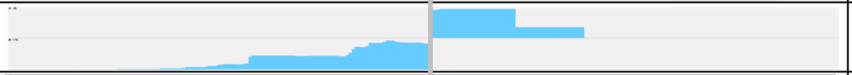	Overlap
chr8:140027813-140027836	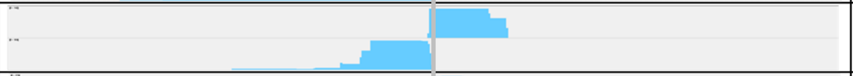	PEACSeq Unique
chr10:103547698-103547721	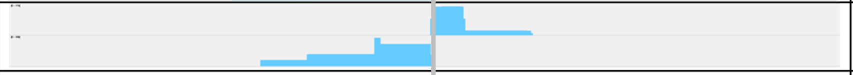	PEACSeq Unique
chr6:156757186-156757209	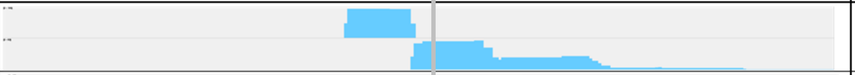	Overlap
chr7:51226564-51226587	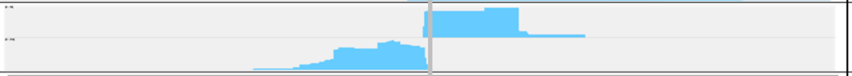	Overlap
chr2:73089915-73089938	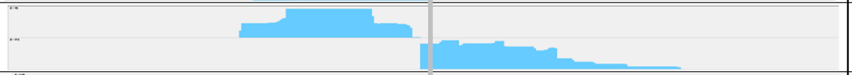	Overlap
chr22:43543399-43543422	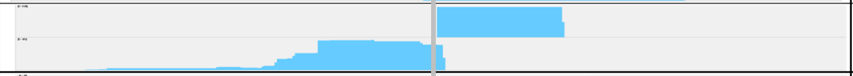	Overlap
chrX:106371167-106371190	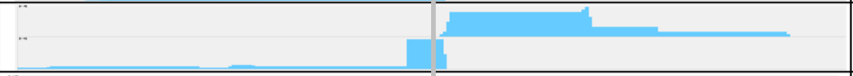	PEACSeq Unique
chr2:229641518-229641541	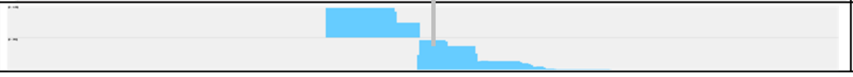	Overlap

Cut site

sFig. 5 | Signal tracks of PEAC-seq at VEGFA TS3

a. The Venn diagram shows the overlap of on-target and off-targets of VEGFA TS3 between the PEAC-seq and GUIDE-seq. Thirty-five sites were overlapped. Twenty-five sites were GUIDE-seq unique, and eight sites were PEAC-seq unique.

b. The GUIDE-seq visualization output of PEAC-seq sites at VEGFA TS3.

c. The signal tracks of PEAC-seq sites at VEGFA TS3. Chromosome locations and the overlap with GUIDE-seq were also shown. Source data are provided as a Source Data file.

Supplementary Fig. 6

a **EMX1**

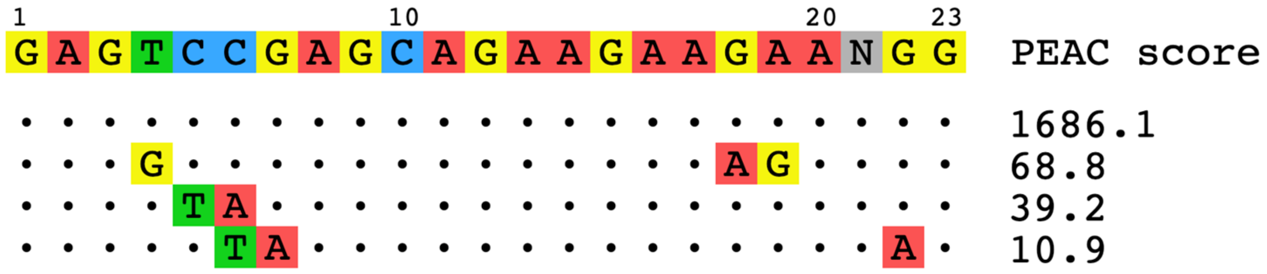

b

Target location (hg38)	EMX1 IGV Screenshot	Compare to GUIDE-seq
chr2:72933852-72933875		Overlap
chr2:218980333-218980356		Overlap
chr5:45358958-45358981		Overlap
chr15:43817548-43817571		Overlap

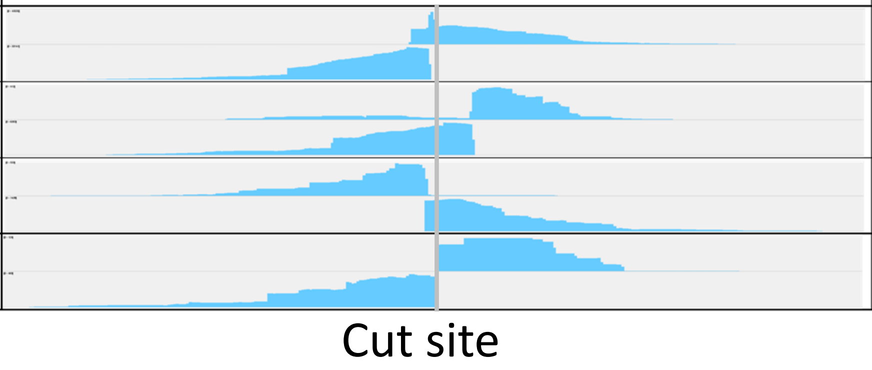

sFig. 6 | Signal tracks of PEAC-seq at EMX1

A. The GUIDE-seq visualization output of PEAC-seq sites at EMX1.

B. The signal tracks of PEAC-seq sites at EMX1. Chromosome locations and the overlap with GUIDE-seq were also shown.

Supplementary Fig. 7

a

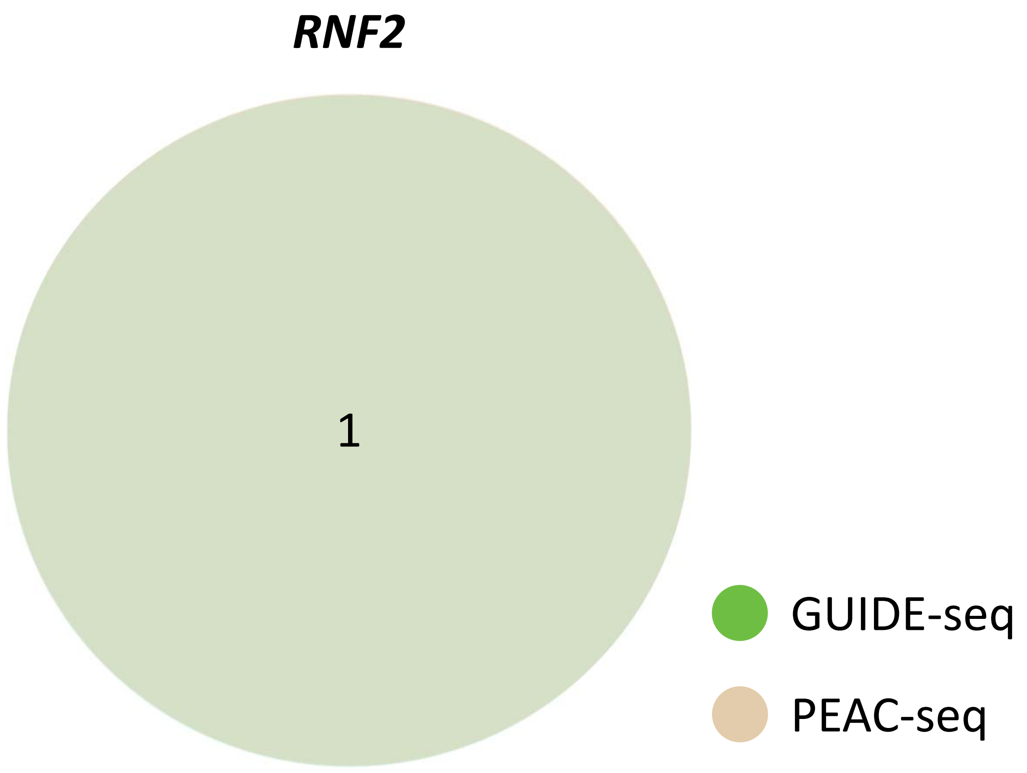

b

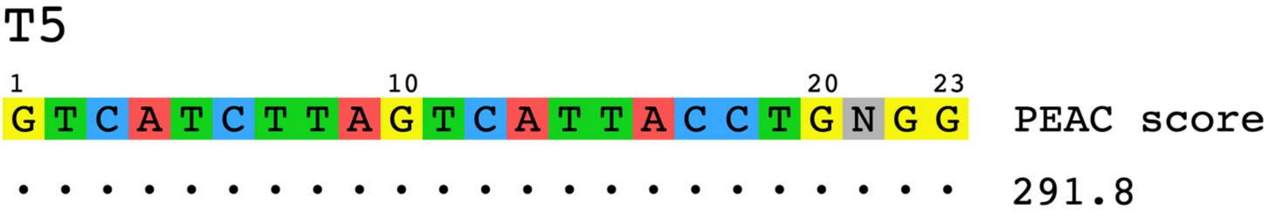

c

Target location (hg38)	RNF2 IGV Screenshot	Compare to GUIDE-seq
chr1:185087634-185087657	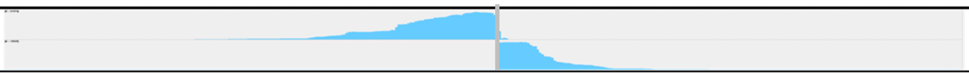	Overlap

Cut site

sFig. 7 | Signal tracks of PEAC-seq at RNF2

a. The Venn diagram shows the overlap of on-target and off-targets of RNF2 between the PEAC-seq and GUIDE-seq. One site was called by both two methods.

b. The GUIDE-seq visualization output of PEAC-seq sites at RNF2.

c. The signal tracks of PEAC-seq sites at RNF2. Chromosome locations and the overlap with GUIDE-seq were also shown.

Supplementary Fig. 8

a **FANCF**

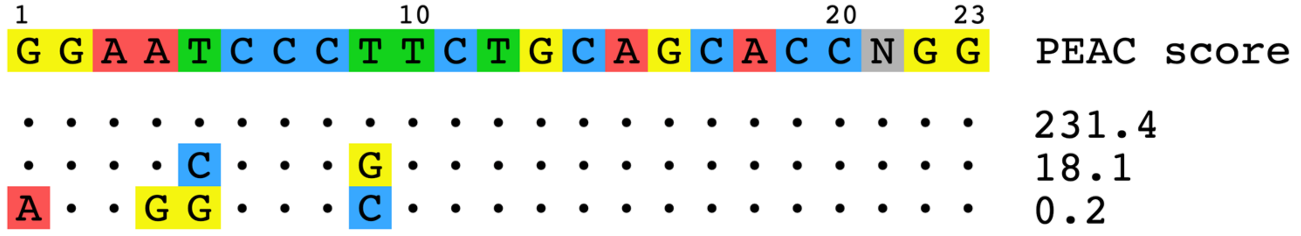

b

Target location (hg38)	FANCF IGV Screenshot	Compare to GUIDE-seq
chr11:22625785-22625808	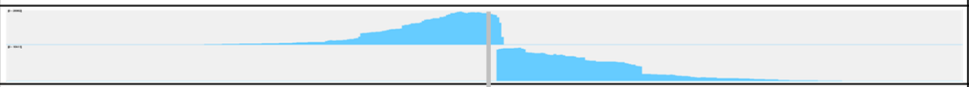	Overlap
chr18:8707523-8707546	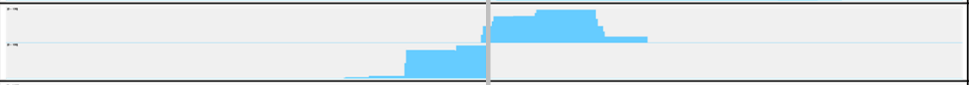	Overlap
chr17:80950160-80950183	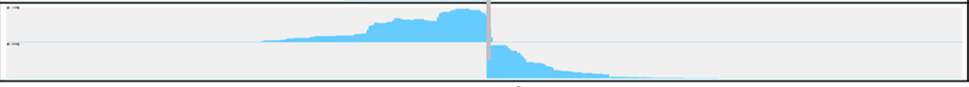	Overlap

Cut site

sFig. 8 | Signal tracks of PEAC-seq at FANCF

a. The GUIDE-seq visualization output of PEAC-seq sites at FANCF.

b. The signal tracks of PEAC-seq sites at FANCF. Chromosome locations and the overlap with GUIDE-seq were also shown. Source data are provided as a Source Data file.

Supplementary Fig. 9

a

Chromosome sites	Off-Target Sequence	primerE.geometric_mean	Translocation Rate
chr6:43769716-43769739	GGTGAGTGAGTGTGTGCGTGTGG	1026.781659	0.002112077
chr14:65102434-65102457	AGTGAGTGAGTGTGTGTGTGGGG	610.920327	0.003035542
chr5:90145145-90145168	AGAGAGTGAGTGTGTGCATGAGG	252.041344	0.003334458
chr5:116098961-116098984	TGTGGGTGAGTGTGTGCGTGAGG	232.609872	0.004450403
chr14:105562677-105562700	GGTGAGTGAGTGTGTGTGTGAGG	136.711319	0.022328863
chr3:194276088-194276111	AGTGAATGAGTGTGTGTGTGTGG	101.254215	0.000355119
chr20:20197633-20197656	AGTGTGTGAGTGTGTGCGTGTGG	80.27332	0.006810514
**chr22:37266776-37266799**	**GCTGAGTGAGTGTATGCGTGTGG**	**66.732341**	**0.347313298**
chr14:73886776-73886799	AGCGAGTGGGTGTGTGCGTGGGG	52.001083	0.051888437
chr12:6827873-6827896	GGTGGATGAGTGTGTGTGTGGGG	51.867834	0.017719232
chr9:23824549-23824572	TGTGGGTGAGTGTGTGCGTGAGA	50.441059	0
chr11:69083657-69083680	GGTGAGTGAGTGCGTGCGGGTGG	49.348584	0.041605582
chr19:6109004-6109027	TGTGAGTGAGTGTGTGTGTGTGA	36.537138	0.062203615
chr19:40055953-40055976	ACTGTGTGAGTGTGTGCGTGAGG	34.844673	0.0042255
**chr14:61612048-61612071**	**TGTGAGTAAGTGTGTGTGTGTGG**	**23.745602**	**0.102367601**
chr22:49344058-49344081	GGTGTGTGAGTGTGTGTGTGTGG	18.189239	0.004552525
chr1:47839359-47839382	TGTGGGTGAGTGTGTGTGTGTGG	17.343618	0
chr1:181588050-181588073	GGAGAGTGAGTGTGTGCATGTGC	15.386906	0
chr2:176598691-176598714	GGTGAGTGTGTGTGTGCATGTGG	13.786543	0
chr4:61201894-61201917	GATGAGTGTGTGTGTGTGTGAGG	12.448211	0
chr7:39301509-39301532	GGTGTGTGAGTGTGTGTGTGTGA	10.217327	0
chr1:212466418-212466441	GGGGAATGAGTGTGTGCATGGAG	9.427523	0
chr19:16458669-16458692	TGTGAGTGAGTGTGTGTGTGGAG	8.036253	0
chr16:79982416-79982439	TGTGAGTGAGTGTGTGCGTGTGA	6.531415	0
chr8:23074983-23075006	TGTGAGTGAGTGTGTGTGTGTGA	6.511907	0
chr8:48085228-48085251	GTAGAGTGAGTGTGTGTGTGTGG	6.307018	0
chr9:18733630-18733653	AGCGAGTGAGTGTGTGTGTGGGG	6.271619	0
chr11:7604557-7604580	GGTGAGTAGGTGTGTGTGTGGGG	5.104442	0
chr6:39060848-39060871	GGTGTGTGAGTGTGTGCATTGGG	4.959486	0
chr10:97000824-97000847	GTTGAGTGAATGTGTGCGTGAGG	4.57412	0
chr3:71583651-71583674	CGCGAGTGAGTGTGTGCGCGGGG	3.907275	0.017795304
chr2:18514941-18514964	AGTGAGAAAGTGTGTGCATGCGG	3.850423	0
chr19:47229228-47229251	CTGGAGTGAGTGTGTGTGTGTGG	3.790482	0.048705569
chr16:83999034-83999057	GGTGAATGAGTGTGTGCTCTGGG	3.52197	0
chr16:12170738-12170761	AGTGAGTGAGTGTGTGTGTGTGA	3.011579	0
chr8:140027813-140027836	AGTGAGTGAGTGTGTGTGTGAAG	2.961645	0
chr10:103547698-103547721	TGAGTGTGAGTGTGTGCGTGGGG	2.508084	0
chr6:156757186-156757209	GATGAGTGAGTGAGTGAGTGGGG	2.258371	0
chr7:51226564-51226587	AGTGAGTAAGTGAGTGAGTGAGG	2.16179	0
chr2:73089915-73089938	GGTGAGTCAGTGTGTGAGTGAGG	2.092342	0
chr22:43543399-43543422	GGTGAGAGAGTGTGTGCACGGGG	1.017226	0
chrX:106371167-106371190	AGTGAATGAGTGTGTGCATGTGA	0.849077	0
chr2:229641518-229641541	GGTGAGCAAGTGTGTGTGTGTGG	0.742496	0

primerE.geometric_mean:
Geometric mean of the number of reads amplified by forward/reverse primer with distinct molecular indices.
Translocation Rate: The ratio of reads amplified by PEAC-seq forward primer but with reverse orientation.

b

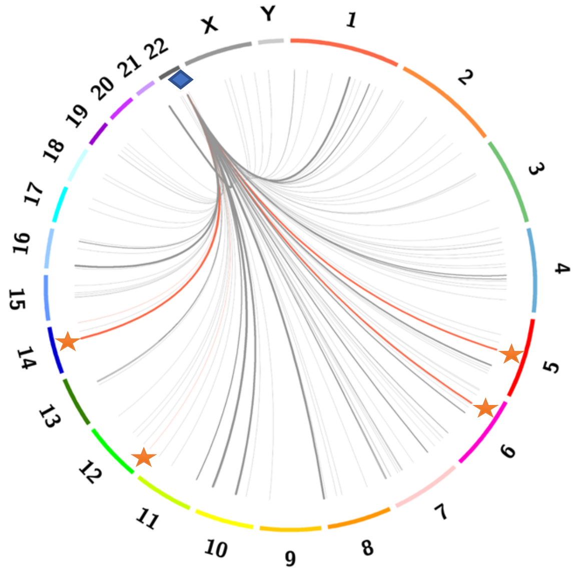

Translocation validation site1

c

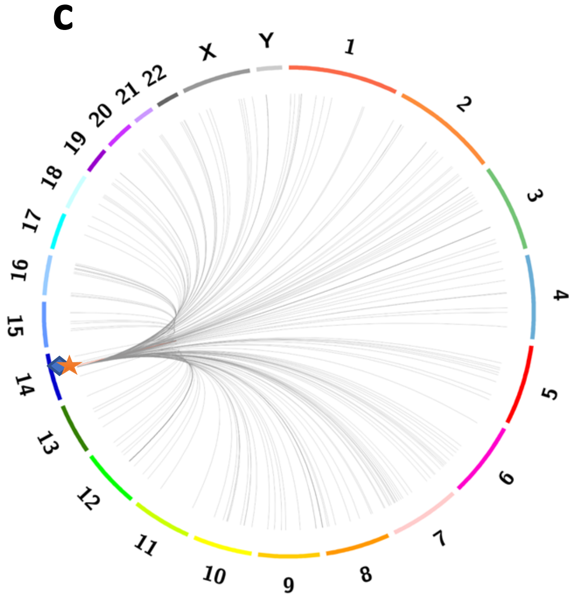

Translocation validation site2

sFig. 9 | Translocations call by PEAC-seq

a. The primerE.geometric_mean and translocation rates of on/off target sites called by PEAC-seq. primerE.geometric_mean: Geometric mean of the number of reads amplified by forward/reverse primer with distinct molecular indices.Translocation Rate: The ratio of reads amplified by PEAC-seq forward primer but with reverse orientation.

b-c. Two of translocation sites with highest translocation rates call by PEAC-seq were validated by unidirectional targeted sequencing (UDiTaS). Circos plots show the chromosome rearrangements at the receiver sites Translocation Validation site1 (chr22:37266776-37266799) **(b)** and Translocation Validation site2 (chr14:61612048-61612071) **(c)**. Both sites are off-targets of VEGFA TS3. The chromosome rearrangements between the Translocation Validation sites and the known VEGFA TS3 off target sites were highlighted by pink arcs and stars. And the rearrangements between the Translocation Validation sites and other undefined sites were shown as grey arcs. Source data are provided as a Source Data file.

Supplementary Fig. 10

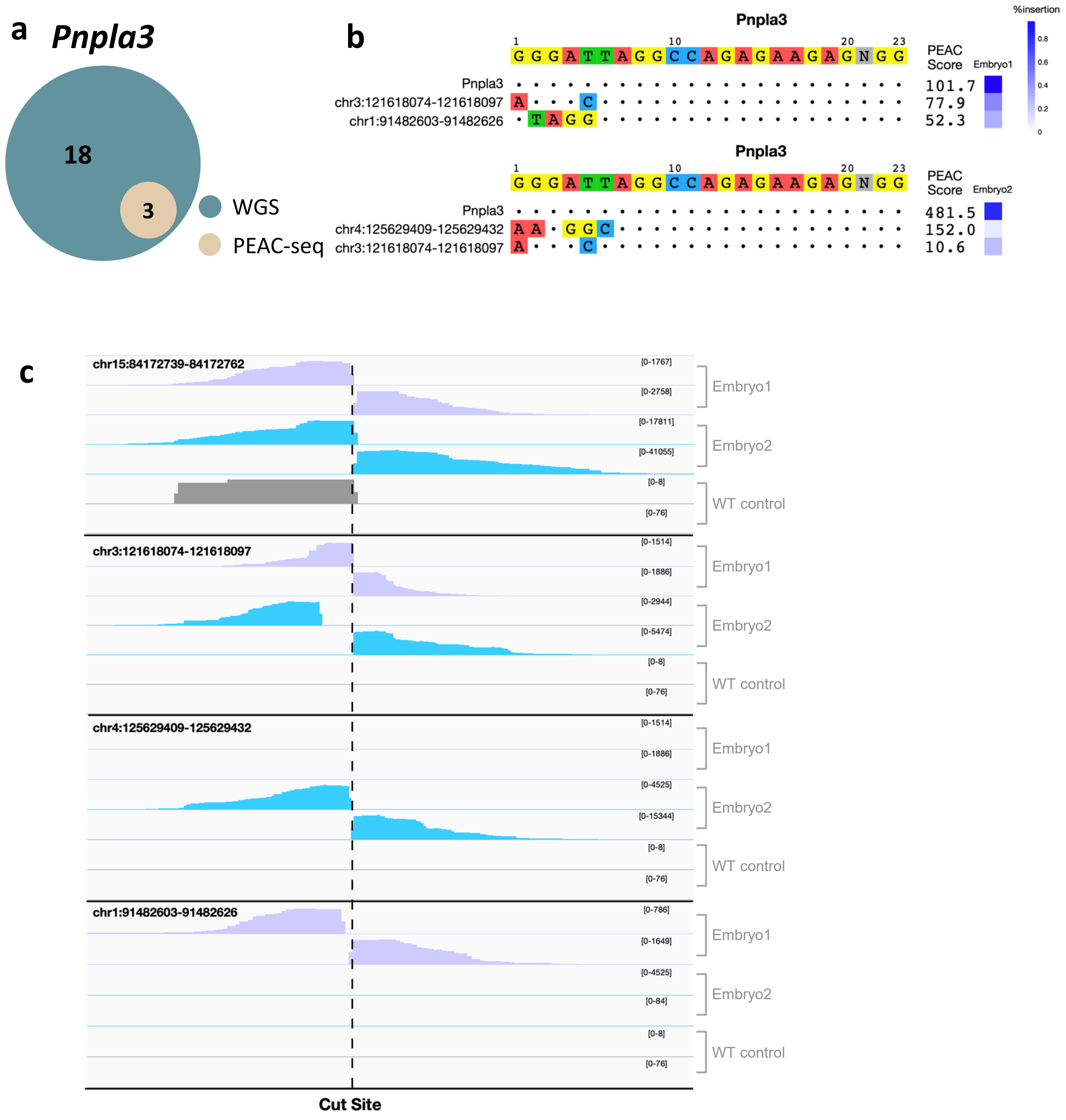

sFig. 10 | PEAC-seq identified mPnpla3 off-targets from edited mouse embryo.

a. The Venn diagram shows the overlap between the PEAC-seq on-target and off-targets of PnPla3 and the top21 off-targets validated from WGS (Anderson et al, 2018). Three cleavage sites were identified from two different embryos from our study. All three sites were reported previously.

b. The sequence visualization of the Pnpla3 on-target and off-targets. One off-target site was identified by both embryos, and each embryo identified an embryo-specific off-target. All three off-targets were reported previously and also verified from Amplicon-NGS.

c. The signal track of the on-target and off-targets sites identified from PEAC-seq in two different embryos and wild-type control. Source data are provided as a Source Data file.

Supplementary Fig. 11

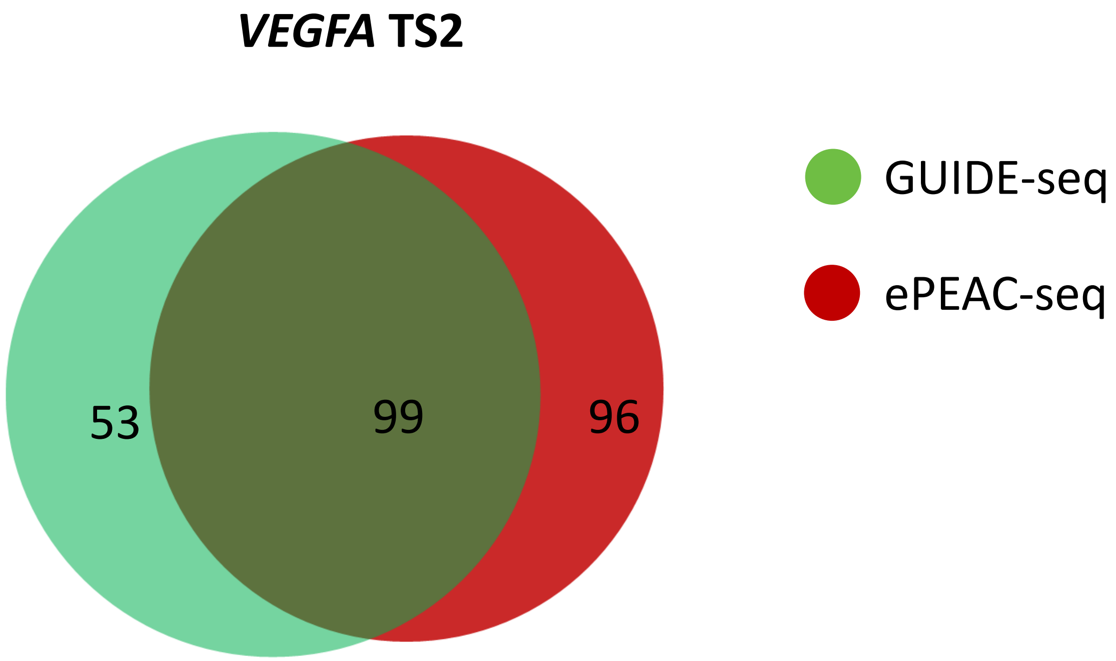

sFig. 11 | ePEAC-seq is an enhanced version of PEAC-seq
The Venn diagram of *VEGFA* TS2 off-targets identified by PEAC-seq and GUIDE-seq. Source data are provided as a Source Data file.

Supplementary Fig. 12

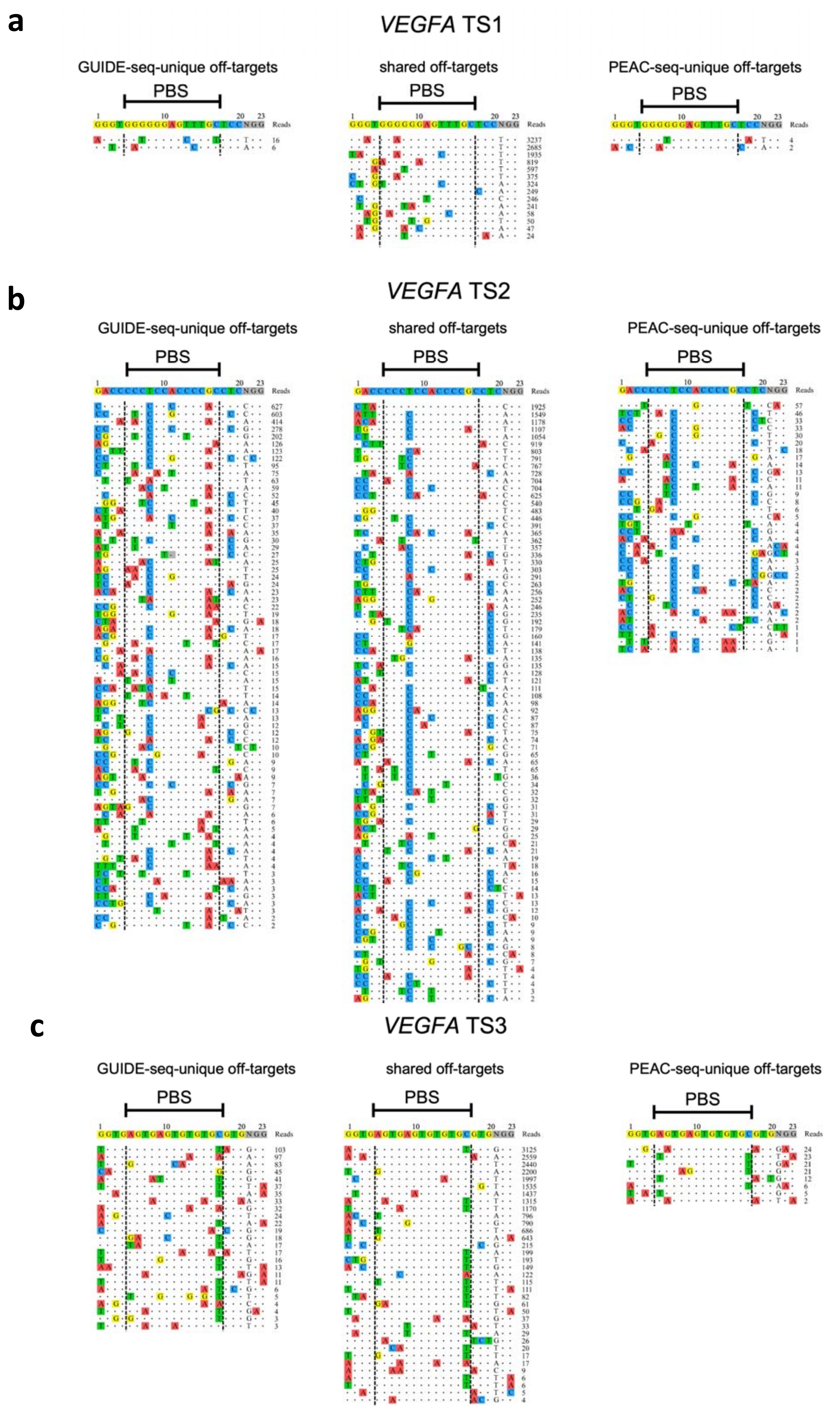

sFig. 12 | PBS mismatches in VEGFA off-targets

a. PBS mismatches in *VEGFA* TS1 off-targets. GUIDE-seq unique and PEAC-seq unique off-targets were verified by Amplicon-seq. **b.** PBS mismatches in *VEGFA* TS2 off-targets. PEAC-seq off-targets were compared to those identified by GUIDE-seq but without Amplicon-seq validation. **c.** PBS mismatches in *VEGFA* TS3 off-targets. PEAC-seq off-targets were compared to those identified by GUIDE-seq but without Amplicon-seq validation.

Supplementary Fig. 13

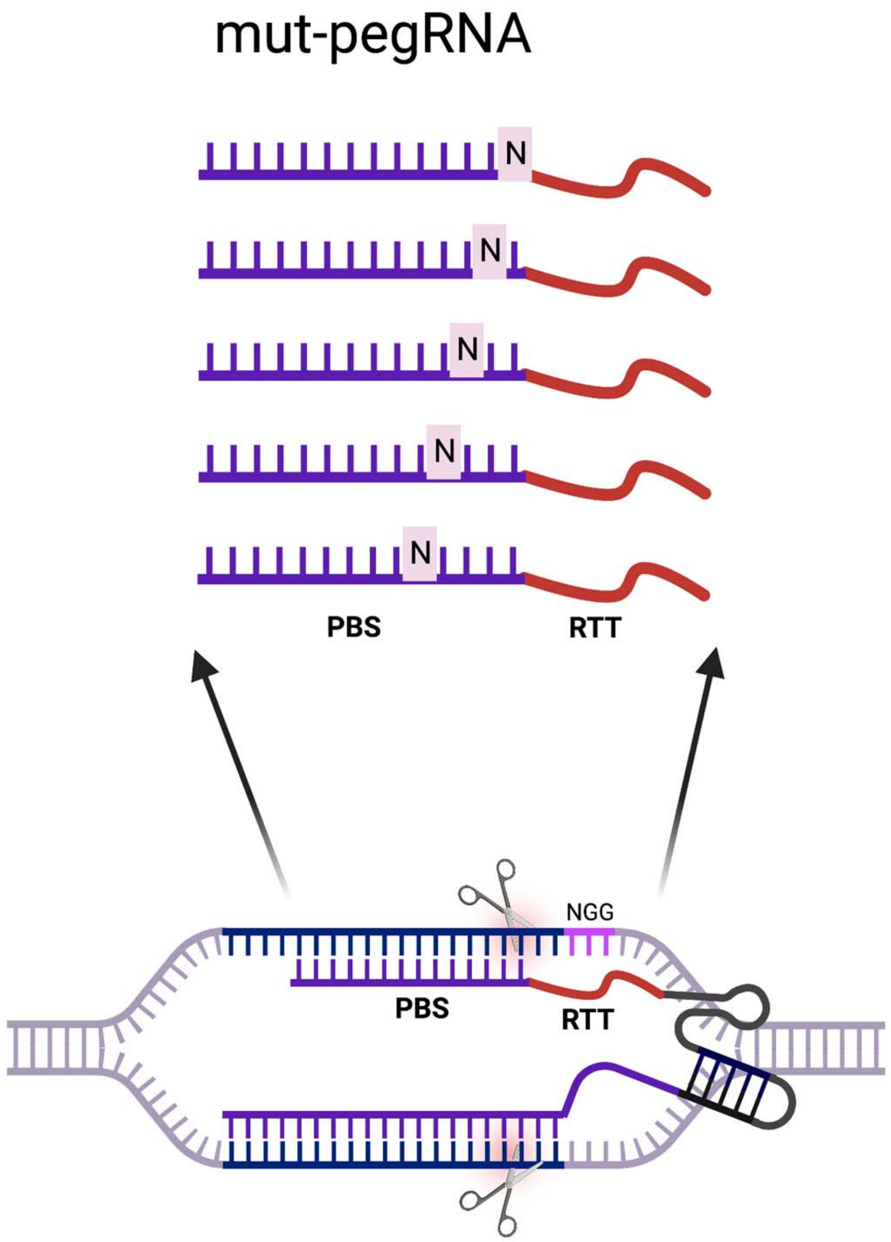

sFig. 13 | Apply mut-pegRNA to increase the insertion efficiency of PEAC-seq tag
Random nucleotide was incorporated into the PBS region of pegRNAs to improve the binding between pegRNA and off-targets with PBS mismatches.

Supplementary Fig. 14

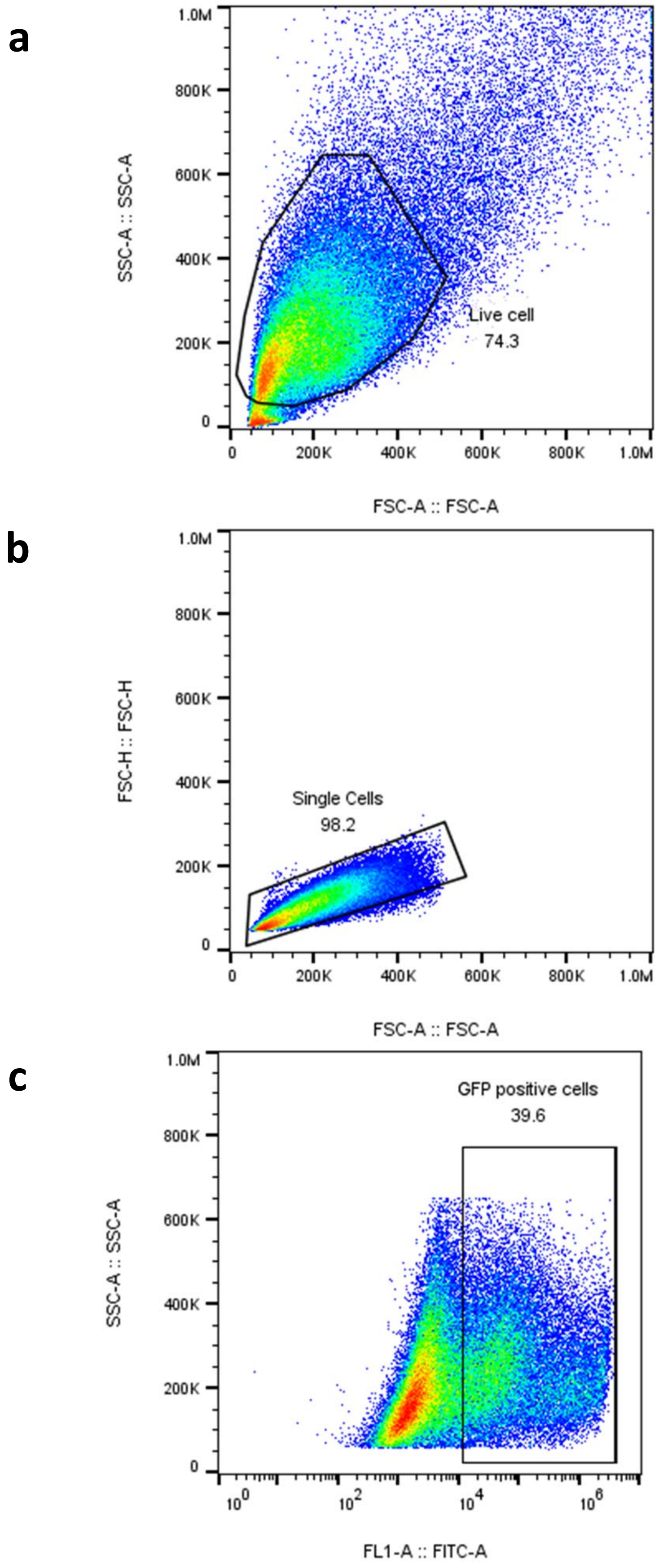

sFig. 14 | PEAC-seq flow cytometry gating strategy

The gating strategy of PEAC-seq in HEK293T cell. **a.** Use FSC-A and SSC-A gate for live cells. **b.** Use FSC-A and FSC-H gate for single cells. **c.** Use SSC-A and FITC-A gate for GFP positive cells. GFP positive cells were sorted for PEAC-seq experiment

Table S1. *In Cellula VEGFA TS1* off-target sites called by PEACSeq 6 set primers

Off-Target Sequence	Mismatches	Length	Off-Target locus	PEAC-seq score	Translocation score
GGGTGGGGGGAGTTTGCTCCTGG	0	23	chr6:43769553-43769576	433.062713	0.004
GGATGGAGGGAGTTTGCTCCTGG	2	23	chr15:65345192-65345215	353.354225	0.010
GGGAGGGTGGAGTTTGCTCCTGG	2	23	chr12:131205636-131205659	202.850933	0.000
GGGGAGGGGAAGTTTGCTCCTGG	3	23	chr1:98882088-98882111	143.171713	0.004
CGGGGGAGGGAGTTTGCTCCTGG	3	23	chr12:1878893-1878916	96.600435	0.028
TAGTGGAGGGAGCTTGCTCCTGG	4	23	chr17:41640069-41640092	77.505772	0.036
GTGGGGGTAGAGTTTGCTCCAGG	4	23	chr6:14316135-14316158	42.573634	0.002
GGGTGGGGGGAGTTTGCCCCAGG	1	23	chr22:36819214-36819237	42.276972	0.004
CTGGTGGGGGAGCTTGCTCCAGG	6	23	chr17:49240170-49240193	10.821438	0.000
GCGTGGGGGGTGTTTGCTCCCGG	2	23	chr5:32945151-32945174	5.823641	0.000
GAGGGGGAGCAGTTTGCTCCAGG	4	23	chr22:19710933-19710956	4.93556	0.000
GGTGGGGGTGGGTTTGCTCCTGG	4	23	chr5:56876245-56876268	3.707808	0.000
GAGTGGGTGGAGTTTGCTACAGG	3	23	chr4:8452058-8452081	2.489623	0.000
GGAGGAGGGGAGTCTGCTCCAGG	4	23	chr1:233021590-233021613	2.425736	0.000
GGGTGGGTGGAGTTTGCTACTGG	2	23	chr1:33177669-33177692	1.620131	0.009
AGCTGGAGGGAGTTTGCCCCAGG	4	23	chr10:122971882-122971905	0.88787	0.000

Table S2. *In Cellula VEGFA TS2* off-target sites called by PEACSeq 6 set primers

Off-Target Sequence	Mismatches	Length	Off-Target locus	PEAC-seq score	Translocation score
GACCCCCTCCACCCCGCCTCCGG	0	23	chr6:43770818-43770841	273.505879	0.010
GGGCCCCTCCACCCCGCCTCTGG	3	23	chr11:31795928-31795951	190.594514	0.002
ACACCCCCCCACCCCGCCTCAGG	4	23	chr9:100837360-100837383	188.074669	0.000
CTACCCCTCCACCCCGCCTCCGG	3	23	chr5:6714988-6715011	152.727719	0.003
GCCCCCACCCACCCCGCCTCTGG	3	23	chr18:23779588-23779611	104.310168	0.021
TACCCCCCACACCCCGCCTCTGG	3	23	chr17:4455450-4455473	100.262218	0.013
GACCCCCCCCACCCCGCCCCCGG	2	23	chr15:32993899-32993922	85.745169	0.003
CGCCCTCCCCACCCCGCCTCCGG	4	23	chr10:133336426-133336449	74.449377	0.052
ATTCCCCCCCACCCCGCCTCAGG	4	23	chr2:241275174-241275197	70.40841	0.016
CTCCCCCCCCTCCCCGCCTCGGG	4	23	chr5:139648654-139648677	64.545795	0.028
TGCCCCTCCCACCCCGCCTCTGG	4	23	chr17:41888497-41888520	62.950212	0.008
CTGCCCCCCCACCCCGCCACTGG	5	23	chrX:150764038-150764061	59.524956	0.038
CTCCCCACCCACCCCGCCTCAGG	4	23	chr4:38535989-38536012	55.626272	0.014
CACTCCCCCCACCCCGCCCCAGG	4	23	chr9:123375895-123375918	52.579827	0.018
GCTTCCCTCCACCCCGCATCCGG	5	23	chr11:72237743-72237766	45.890801	0.018
CCTCCCCCACACCCCGCATCCGG	6	23	chr1:151059392-151059415	38.532438	0.051
GTACCCCACCACCCCGCCCCAGG	5	23	chr8:143740774-143740797	38.030442	0.038
CCCCCCCCCCCCCCCGCCTCCGG	4	23	chr22:50446356-50446379	31.239449	0.029
GACCCTGTCCACCCCACCTCAGG	3	23	chr1:939940-939963	31.237905	0.028
GACTCCCTCCGCCCCGCTTCCAG	4	23	chr5:180161284-180161307	25.959391	0.064
CATACCCCCCACCCCGCCCCGGG	6	23	chr4:8840184-8840207	25.699925	0.024
AAGCCCCCCCACCCCGCCCCGGG	4	23	chr9:137368986-137369009	24.144971	0.004
CCCCACCCCCACCCCGCCTCAGG	4	23	chr10:114534490-114534513	23.876483	0.000
GAGCTCCCCCACCCCGCCCCGGG	4	23	chr19:14032156-14032179	22.162141	0.089
CCACCCCCCCACCCCGCCCCTGG	5	23	chr20:10933306-10933329	19.602157	0.000
AGGCCCCCACACCCCGCCTCAGG	5	23	chr4:1494513-1494536	17.757267	0.012
CCACCCCCCCACCCCGCCCCAGG	5	23	chr2:169716823-169716846	17.494795	0.001
GGCTCCCTCCGCCCCGCCCCGGG	4	23	chr11:65712291-65712314	17.255456	0.000
CCCCCCTCCCACCCCGCCTCTAG	5	23	chr9:37465350-37465373	16.894989	0.000
TGCCCCCCCCACCCCGCCCCCGG	4	23	chrX:129906647-129906670	16.804222	0.000
CCCCCCCCCCACCCCGCCCTCGG	5	23	chr11:19777094-19777117	16.532662	0.000
CCGCCCCTCCACCCCGCCACTGG	4	23	chr17:57663161-57663184	16.447721	0.000
CCGCCCCCCCACCCCGCCGCCGG	5	23	chr5:171451048-171451071	16.344224	0.000
CAACCCCCCCACCCCGCTTCAGG	4	23	chr3:140679941-140679964	16.001981	0.000
TCCCCCCCACCCCCGACCTCAGG	7	23	chr13:26052087-26052110	15.113945	0.000
GACCCCTCACACCCCGCCCCTGG	4	23	chr7:95690358-95690381	14.501028	0.003
CCCCCCCCCCACCCCGCCCCCGG	4	23	chr2:128486621-128486644	14.443977	0.023
TCTCCACCCCACCCCGCCCCTGG	6	23	chr1:77779744-77779767	13.679351	0.002
ACCCCCCCCCGCCCCGCCCCCGG	5	23	chr11:73747435-73747458	13.372696	0.086
GACCCGCCCCGCCCCGCCTCTGG	3	23	chr1:11654471-11654494	12.693298	0.000
CCCCCACCCCACCCCGCCTCCAG	5	23	chr9:127900790-127900813	12.514422	0.000
CTCCCCCGCCACCCCGCCCCAGG	4	23	chr17:17051531-17051554	12.244711	0.000
CCCCCCCACCACCCCGCCCCGGG	4	23	chr8:18184030-18184053	10.42162	0.000
GTCCCTCCCCACCCCGCCTTGGG	4	23	chr22:39706378-39706401	10.415638	0.007
CCCCCCCCCCCCCCCGCCTCAGG	4	23	chr13:99894728-99894751	9.983946	0.000
GCCCCCCACCACCCCACCTCGGG	3	23	chr19:13011357-13011380	9.555546	0.000
TCCACCCCCCACCCCGCCCCGGG	5	23	chr7:105293491-105293514	9.25911	0.002
CTCCCCCTCCACCCCACCTCCAG	4	23	chr6:31495043-31495066	8.683129	0.000
GCCACCCCCCACCCCGCCTCTGC	4	23	chr6:98825031-98825054	8.103262	0.000
AGGCCCCCCCGCCCCGCCTCAGG	5	23	chr11:374660-374683	7.837553	0.015
CACCACCCCCACCCCGCCCCTGG	4	23	chr3:195762332-195762355	7.175532	0.000
GACCCCTCCCACCCCGACTCCGG	3	23	chr9:27338859-27338882	6.33333	0.024
CCCCCCCCTCACCCCGCCCCTGG	5	23	chr19:44821307-44821330	5.855975	0.012
GGCCCCACCCACCCCGCCGCAGG	4	23	chr19:29845075-29845098	5.768108	0.000
AGCCCCCACCTCCCCGCCTCGGG	4	23	chr22:43288472-43288495	5.66825	0.000
TGCCCCCCCCACCCCACCTCTGG	4	23	chr16:56929510-56929533	4.990966	0.026
GACACCTTCCACCCCGTCTCTGG	3	23	chr11:63623611-63623634	4.978597	0.000
GACCCCTCCCCCCCCACCTCAGG	4	23	chr12:57087439-57087462	4.794541	0.000
TGCACCCCCCACCCCGCCCCTGG	5	23	chr5:133524667-133524690	4.508989	0.000
CCCCCCCCCCACCCCGCCCCGAG	5	23	chr12:22334709-22334732	4.472575	0.000
CCCCCCTCCCACCCCGCCCCGGG	5	23	chr1:26696329-26696352	4.400671	0.000
AACCCCACCCACCCCACCCCCGG	5	23	chr8:103500163-103500186	4.347504	0.000
GACCCCTCCCTCCCCACCTCAGG	4	23	chr1:50976513-50976536	4.185232	0.000
TTCTCCCTCCTCCCCGCCTCGGG	4	23	chr19:42302642-42302665	4.110585	0.097
CCCCACCCCCACCCCGCCCCCGG	5	23	chr3:38099767-38099790	3.658016	0.000
CTACCCCCACTCCCCGCCTCCGG	6	23	chr10:101061743-101061766	3.359073	0.047
GATCGACTCCACCCCGCCTCTGG	3	23	chr12:31659398-31659421	3.013712	0.000
AAGACCCCCCACCCCGCCCCAGG	6	23	chr19:45448779-45448802	2.930733	0.000
ACTCCCCTCCACCCCACCTCTGA	5	23	chr1:23435751-23435774	2.799268	0.000
CAGTCCCCCCACCCCACCTCTGG	6	23	chr10:70778444-70778467	2.613543	0.000
CGTCCCCCCCCCCCCGCCTCAGG	5	23	chr21:45283300-45283323	2.60841	0.000
CCGCCACCCCACCCCGCCCCCGG	6	23	chr15:84748852-84748875	2.593206	0.000
CCCCCCCCCCGCCCCGCCTCCAG	5	23	chr10:100969482-100969505	2.538089	0.000
ATCCCCCTCCACCCCACCCCTGG	4	23	chr11:12287370-12287393	2.348836	0.000
CACCCCGCCCACCCCGCCCCTGG	4	23	chr8:37393659-37393682	2.250924	0.000
CACCCCCCCCCCCCCGCCCCCGG	4	23	chr17:81923260-81923283	2.226677	0.000
GCCCCCCCCAACCCCGCCCCCGG	4	23	chr4:7327802-7327825	2.185666	0.000
CATCCCCCCCACCCCACCCCGGG	5	23	chr11:46120297-46120320	2.08909	0.000
GTCCCCTCCCTCCCCGCCTCTGG	4	23	chr9:33202609-33202632	2.006285	0.000
TCTCCCCTCCACCCCGCCCTCGG	5	23	chr11:133907545-133907568	1.976671	0.000
ACCCCCCCCCCCCCCGCCCCCGG	5	23	chr2:24634707-24634730	1.974975	0.000
CACCCCCCGCACCCCGCCCCAGG	4	23	chr11:32162932-32162955	1.953479	0.000
TGTCCCCTCTACCCCGCTTCAGG	5	23	chr11:72750152-72750175	1.796193	0.000
CCTCCCCAACACCCCGCCCCGGG	6	23	chr2:47570989-47571012	1.673741	0.000
AACCACCCCCACCCCACCCCAGG	5	23	chr7:50697814-50697837	1.67294	0.000
ACCACCCCCCCCCCCGCCCCAGG	6	23	chr19:58154691-58154714	1.560603	0.000
TACCCCCCCCACCCCGCCACAGG	3	23	chr11:13926806-13926829	1.528055	0.000
CTTCCCCCACACCCCGCCCCAGG	6	23	chr3:31417159-31417182	1.518376	0.000
GTCACTCCCCACCCCGCCTCTGG	4	23	chr11:83722550-83722573	1.501509	0.000
CCCCACCCCCACCCCATCTCTGG	6	23	chr19:18020497-18020520	1.373777	0.000
AACACCCCCCACCCCACCCCGGG	5	23	chr19:51412258-51412281	1.360049	0.000
CCCACCCCCCACCCCGCCCCAGG	5	23	chr9:133572178-133572201	1.341193	0.000
AGCCCCCCCCTCCCCGCCCCAGG	5	23	chr14:104134854-104134877	1.225248	0.000
CCCCCCCCCCCCCCCGCCCCAGG	5	23	chr13:51767773-51767796	1.216465	0.000
GCCCCCCCCCCCCCCGCCCGGCC	7	23	chr2:222559160-222559183	1.194224	0.000
GACCCCCCCCCCCCCGCCCCCGGG	5	23	chr3:13145795-13145818	1.166401	0.000
TGCCCCCCCCACCCCCCCTACAGG	6	23	chr2:169967228-169967251	1.139076	0.000
GCCGCCCCCCACTCCGCCTCCGG	4	23	chr4:142846213-142846236	1.0873	0.000
CACCCCCTCCCCTCCGCCTCAGG	3	23	chr11:61553960-61553983	0.996607	0.000
CCGCCCCCCCATCCCGCCCCAGG	6	23	chr20:63944457-63944480	0.982384	0.000
ACCCCCCCCCCACCCGCCCCCGG	6	23	chr6:2737969-2737992	0.9617	0.000
CAACACCCCCACCCCGCCTCACA	6	23	chr12:68807995-68808018	0.927647	0.000
AACCTCCCCCACCCCACCCCAGG	5	23	chr17:48026474-48026497	0.89679	0.000
CTCCGCCCCCACCCCGCCCCCGG	5	23	chr1:27235142-27235165	0.866381	0.000
TGCCCCCTCCACCCCACCTCTGA	4	23	chr6:110199814-110199837	0.78693	0.000
GCCCCCTCCCACCCCGCCCCAAG	5	23	chr2:66556906-66556929	0.74852	0.000
ACTCCCCTCCACCCCGGCTCGGG	4	23	chr17:64089687-64089710	0.69465	0.000
CAACCTCTCCACCCCGCCGAGCT	7	23	chr16:8403326-8403349	0.665077	0.000
GTCCCCTCCCACCCCGCCTCCAG	4	23	chr14:89936960-89936983	0.640436	0.004
CCCCACCTCCACCCCCCTCTCCTT	7	23	chr8:67343376-67343399	0.590339	0.000
TTCCACCCCCACCCCGCCTCAGA	5	23	chr15:74690775-74690798	0.528016	0.000
ACCCCCCACCCCCCAACCTCAGC	7	23	chr2:239424835-239424858	0.390902	0.000
GTCTCCCACCCCCCAACCTCGGG	6	23	chr20:2321725-2321748	0.329502	0.000
TCCACCCACCCCCCAACCTCAGG	7	23	chr14:25322642-25322665	0.269037	0.000
ATCCCCCTCCAACCCGCTCCAGG	5	23	chr1:205749914-205749937	0.258881	0.000

Table S3. *In Cellula VEGFA TS3* off-target sites called by PEACSeq 6 set primers

Off-Target Sequence	Mismatches	Length	Off-Target locus	PEAC-seq score	Translocation score
GGTGAGTGAGTGTGTGCGTGTGG	0	23	chr6:43769716_43769739	1026.781659	0.002
AGTGAGTGAGTGTGTGTGTGGGG	2	23	chr14:65102434_65102457	610.920327	0.003
AGAGAGTGAGTGTGTGCATGAGG	3	23	chr5:90145145_90145168	252.041344	0.003
TGTGGGTGAGTGTGTGCGTGAGG	2	23	chr5:116098961_116098984	232.609872	0.004
GGTGAGTGAGTGTGTGTGTGAGG	1	23	chr14:105562677_105562700	136.711319	0.022
AGTGAATGAGTGTGTGTGTGTGG	3	23	chr3:194276088_194276111	101.254215	0.000
AGTGTGTGAGTGTGTGCGTGTGG	2	23	chr20:20197633_20197656	80.27332	0.007
GCTGAGTGAGTGTATGCGTGTGG	2	23	chr22:37266776_37266799	66.732341	0.347
AGCGAGTGGGTGTGTGCGTGGGG	3	23	chr14:73886776_73886799	52.001083	0.052
GGTGGATGAGTGTGTGTGTGGGG	3	23	chr12:6827873_6827896	51.867834	0.018
TGTGGGTGAGTGTGTGCGTGAGA	3	23	chr9:23824549_23824572	50.441059	0.000
GGTGAGTGAGTGCGTGCGGGTGG	2	23	chr11:69083657_69083680	49.348584	0.042
TGTGAGTGAGTGTGTGTGTGTGA	3	23	chr19:6109004_6109027	36.537138	0.062
ACTGTGTGAGTGTGTGCGTGAGG	3	23	chr19:40055953_40055976	34.844673	0.004
TGTGAGTAAGTGTGTGTGTGTGG	3	23	chr14:61612048_61612071	23.745602	0.102
GGTGTGTGAGTGTGTGTGTGTGG	2	23	chr22:49344058_49344081	18.189239	0.005
TGTGGGTGAGTGTGTGTGTGTGG	3	23	chr1:47839359_47839382	17.343618	0.000
GGAGAGTGAGTGTGTGCATGTGC	3	23	chr1:181588050_181588073	15.386906	0.000
GGTGAGTGTGTGTGTGCATGTGG	2	23	chr2:176598691_176598714	13.786543	0.000
GATGAGTGTGTGTGTGTGTGAGG	3	23	chr4:61201894_61201917	12.448211	0.000
GGTGTGTGAGTGTGTGTGTGTGA	3	23	chr7:39301509_39301532	10.217327	0.000
GGGGAATGAGTGTGTGCATGGAG	4	23	chr1:212466418_212466441	9.427523	0.000
TGTGAGTGAGTGTGTGTGTGGAG	3	23	chr19:16458669_16458692	8.036253	0.000
TGTGAGTGAGTGTGTGCGTGTGA	2	23	chr16:79982416_79982439	6.531415	0.000
TGTGAGTGAGTGTGTGTGTGTGA	3	23	chr8:23074983_23075006	6.511907	0.000
GTAGAGTGAGTGTGTGTGTGTGG	4	23	chr8:48085228_48085251	6.307018	0.000
AGCGAGTGAGTGTGTGTGTGGGG	3	23	chr9:18733630_18733653	6.271619	0.000
GGTGAGTAGGTGTGTGTGTGGGG	3	23	chr11:7604557_7604580	5.104442	0.000
GGTGTGTGAGTGTGTGCATTGGG	3	23	chr6:39060848_39060871	4.959486	0.000
GTTGAGTGAATGTGTGCGTGAGG	2	23	chr10:97000824_97000847	4.57412	0.000
CGCGAGTGAGTGTGTGCGCGGGG	3	23	chr3:71583651_71583674	3.907275	0.018
AGTGAGAAAGTGTGTGCATGCGG	4	23	chr2:18514941_18514964	3.850423	0.000
CTGGAGTGAGTGTGTGTGTGTGG	4	23	chr19:47229228_47229251	3.790482	0.049
GGTGAATGAGTGTGTGCTCTGGG	4	23	chr16:83999034_83999057	3.52197	0.000
AGTGAGTGAGTGTGTGTGTGTGA	3	23	chr16:12170738_12170761	3.011579	0.000
AGTGAGTGAGTGTGTGTGTGAAG	3	23	chr8:140027813_140027836	2.961645	0.000
TGAGTGTGAGTGTGTGCGTGGGG	3	23	chr10:103547698_103547721	2.508084	0.000
GATGAGTGAGTGAGTGAGTGGGG	3	23	chr6:156757186_156757209	2.258371	0.000
AGTGAGTAAGTGAGTGAGTGAGG	4	23	chr7:51226564_51226587	2.16179	0.000
GGTGAGTCAGTGTGTGAGTGAGG	2	23	chr2:73089915_73089938	2.092342	0.000
GGTGAGAGAGTGTGTGCACGGGG	3	23	chr22:43543399_43543422	1.017226	0.000
AGTGAATGAGTGTGTGCATGTGA	4	23	chrX:106371167_106371190	0.849077	0.000
GGTGAGCAAGTGTGTGTGTGTGG	3	23	chr2:229641518_229641541	0.742496	0.000

Table S4. *In Cellula EMX1* off-target sites called by PEACSeq 6 set primers

Off-Target Sequence	Mismatches	Length	Off-Target locus	PEAC-seq score	Translocation score
GAGTCCGAGCAGAAGAAGAAGGG	0	23	chr2:72933852-72933875	1477.013219	0.000
GAGGCCGAGCAGAAGAAAGACGG	3	23	chr2:218980333-218980356	61.01071	0.049
GAGTTAGAGCAGAAGAAGAAAGG	2	23	chr5:45358958-45358981	29.528328	0.017
GAGTCTAAGCAGAAGAAGAAGAG	3	23	chr15:43817548-43817571	9.271507	0.000

Table S5. *In Cellula RNF2* off-target sites called by PEACSeq 6 set primers

Off-Target Sequence	Mismatches	Length	Off-Target locus	PEAC-seq score	Translocation score
GTCATCTTAGTCATTACCTGAGG	0	23	chr1:185087634-185087657	277.783224	0.001

Table S6. *In Cellula FANCF* off-target sites called by PEACSeq 6 set primers

Off-Target Sequence	Mismatches	Length	Off-Target locus	PEAC-seq score	Translocation score
GGAATCCCTTCTGCAGCACCTGG	0	23	chr11:22625785-22625808	365.548179	0.001
GGAACCCCGTCTGCAGCACCAGG	2	23	chr18:8707523-8707546	39.346062	0.005
AGAGGCCCCTCTGCAGCACCAGG	4	23	chr17:80950160-80950183	2.37021	0.000

Table S7. In vivo *PCSK9* off-target sites called by PEACSeq 6 set primers

**Embryo#5**					
Off-Target Sequence	Mismatches	Length	Off-Target locus	PEAC-seq score	Translocation score
AGCAGCAGCGGCGGCAACAGCGG	0	23	chr4:106463845-106463868	527.607594	0.005
AGCAGCAGCCGCGGCAACAGTGG	1	23	chr6:87111978-87112001	0.343888	0.000
**Embryo#12**					
Off-Target Sequence	Mismatches	Length	Off-Target locus	PEAC-seq score	Translocation score
AGCAGCAGCGGCGGCAACAGCGG	0	23	chr4:106463845-106463868	279.668515	0.001

Table S8. In vivo *Pnpla3* off-target sites called by PEACSeq 6 set primers

**Embryo #21**					
Off-Target Sequence	Mismatches	Length	Off-Target locus	PEAC-seq score	Translocation score
GGGATTAGGCCAGAGAAGAGGGG	0	23	chr15:84172739-84172762	481.527428	0.012
AAGGGCAGGCCAGAGAAGAGGGG	6	23	chr4:125629409-125629432	151.957705	0.000
AGGACTAGGCCAGAGAAGAGAGG	2	23	chr3:121618074-121618097	10.591849	0.000
**Embryo #31**					
Off-Target Sequence	Mismatches	Length	Off-Target locus	PEAC-seq score	Translocation score
GGGATTAGGCCAGAGAAGAGGGG	0	23	chr15:84172739-84172762	101.67443	0.000
AGGACTAGGCCAGAGAAGAGAGG	2	23	chr3:121618074-121618097	77.911256	0.000
GTAGGTAGGCCAGAGAAGAGAGG	5	23	chr1:91482603-91482626	52.333422	0.000

Table S9. Primers used in the validation of chromosome translocation

	Names	Sequences
**Primers for translocation validation site1**	VEGFA3 TS3 Translocation 1 validation F1	GTTGTCAGGTGTAGGGGTTGG
	VEGFA3 TS3 Translocation 1 validation F2	GTGACTGGAGTTCAGACGTGTGCTCTTCCGATCTNNNNNNGGCGACCTCTGTGTACACTG
**Primers for translocation validation site2**	VEGFA3 TS3 Translocation 2 validation F1	TTGCAGAAGCAGGAGATGTTTG
	VEGFA3 TS3 Translocation 2 validation F2	GTGACTGGAGTTCAGACGTGTGCTCTTCCGATCTNNNNNNTCCTAAGCTGTATGTGAGTCCCT

Table S10. Amplicon-seq validation of VEGFA ts1,EMX1,FANCF on/off targets

Site No.	sgRNA	Chromosome	Start	End	PEAC-seq	GUDIE-seq	Edit efficiency (Amplicon-seq)
VEGFA ts1-1	GGGTGGGGGGAGTTTGCTCCTGG	chr6	43769497	43769655	yes	yes	yes(53%)
VEGFA ts1-2	GGATGGAGGGAGTTTGCTCCTGG	chr15	65345100	65345227	yes	yes	yes(40%)
VEGFA ts1-3	GGGAGGGTGGAGTTTGCTCCTGG	chr12	131205651	131205696	yes	yes	yes(6.7%)
VEGFA ts1-4	GGGGAGGGGAAGTTTGCTCCTGG	chr1	98882063	98882097	yes	yes	yes(21%)
VEGFA ts1-5	CGGGGGAGGGAGTTTGCTCCTGG	chr12	1878899	1878938	yes	yes	yes(9%)
VEGFA ts1-6	TAGTGGAGGGAGCTTGCTCCTGG	chr17	41640033	41640116	yes	yes	yes(19.16%)
VEGFA ts1-7	GTGGGGGTAGAGTTTGCTCCAGG	chr6	14316124	14316144	yes	yes	yes(1.09%)
VEGFA ts1-8	GGGTGGGGGGAGTTTGCCCCAGG	chr22	36819211	36819244	yes	yes	yes(0.37%)
VEGFA ts1-9	CTGGTGGGGGAGCTTGCTCCAGG	chr17	49240174	49240186	yes	yes	yes(0.63%)
VEGFA ts1-10	GCGTGGGGGGTGTTTGCTCCCGG	chr5	32945166	32945172	yes	yes	yes(0.9%)
VEGFA ts1-11	GAGGGGGAGCAGTTTGCTCCAGG	chr22	19710933	19710950	yes	yes	yes(0.63%)
VEGFA ts1-12	GGTGGGGGTGGGTTTGCTCCTGG	chr5	56876243	56876254	yes	yes	yes(0.21%)
VEGFA ts1-13	GAGTGGGTGGAGTTTGCTACAGG	chr4	8452074	8452082	yes	yes	yes(0.24%)
VEGFA ts1-14	GGAGGAGGGGAGTCTGCTCCAGG	chr1	233021610	233021621	yes	yes	yes(0.45%)
VEGFA ts1-15	GGGTGGGTGGAGTTTGCTACTGG	chr1	33177670	33177688	yes	no	yes(0.27%)
VEGFA ts1-16	AGCTGGAGGGAGTTTGCCCCAGG	chr10	122971900	122971905	yes	no	yes(1%)
VEGFA ts1-17	GGGCAAGGGGAGGTTGCTCCTGG	chr11	117610486	117610509	no	yes	no(0.27%)
VEGFA ts1-18	AGGAAGGAGGAGTTAGCTCCTGG	chr11	67806773	67806796	no	yes	no(0.2%)
VEGFA ts1-19	AAGTAAGGGAAGTTTGCTCCTGG	chr16	8669349	8669372	no	yes	no(0.16%)
VEGFA ts1-20	GGTGGGGGAGAGCTAGCTCCGGG	chr3	196144386	196144409	no	yes	no(0.01%)
VEGFA ts1-21	AGGAAGGAGGAGTTAGCTCCTGG	chr3	125915142	125915165	no	yes	no(0.25%)
VEGFA ts1-22	GAGGGTGGGGAGTTTACTCCTGG	chr5	7067028	7067051	no	yes	no(0.05%)
VEGFA ts1-23	AGGTGGTGGGAGCTTGTTCCTGG	chr3	128565471	128565494	no	yes	yes(1.55%)
VEGFA ts1-24	GGTTGAGGGGAGTCTGCTCCAGG	chr13	25628656	25628679	no	yes	yes(0.5%)
EMX1-1	GAGTCCGAGCAGAAGAAGAAGGG	chr2	72933852	72933875	yes	yes	yes(77%)
EMX1-2	GAGTTAGAGCAGAAGAAGAAAGG	chr5	45358958	45358981	yes	yes	yes(6%)
EMX1-3	GAGTCTAAGCAGAAGAAGAAGAG	chr15	43817548	43817571	yes	yes	yes(17.2%)
EMX1-4	GAGGCCGAGCAGAAGAAAGACGG	chr2	219000000	218980356	yes	yes	yes(1.1%)
EMX1-5	GAGTCCTAGCAGGAGAAGAAGAG	chr8	128000000	127789018	yes(epeg)	yes	yes(3%)
EMX1-6	AAGTCTGAGCACAAGAAGAATGG	chr5	9227033	9227056	no	yes	no
EMX1-7	GAGTCCGGGAAGGAGAAGAAAGG	chrX	53440757	53440780	yes(epeg)	yes	yes(2.01%)
EMX1-8	GAGCCGGAGCAGAAGAAGGAGGG	chr5	147000000	147453643	no	yes	no(0.29%)
EMX1-9	AAGTCCGAGGAGAGGAAGAAAGG	chr1	23394118	23394141	no	yes	no(0.21%)
EMX1-10	GAATCCAAGCAGGAGAAGAAGGA	chr3	4989912	4989935	no	yes	no(0.2%)
EMX1-11	ACGTCTGAGCAGAAGAAGAATGG	chr6	9118559	9118582	no	yes	no(0.4%)
EMX1-12	GAGTAGGAGCAGGAGAAGAAGGA	chr13	27195503	27195526	no	yes	no(0.1%)
EMX1-13	AAGTCCCGGCAGAGGAAGAAGGG	chr15	99752256	99752279	no	yes	no(0.16%)
EMX1-14	TCATCCAAGCAGAAGAAGAAGAG	chr3	95971335	95971358	no	yes	no
EMX1-15	GAGTCTAAGCAGGAGAATAAAGG	chr2	218000000	217513401	no	yes	no
EMX1-16	GAGCACGAGCAAGAGAAGAAGGG	chr10	57088951	57088974	no	yes	no
FANCF-1	GGAATCCCTTCTGCAGCACCTGG	chr11	22625785	22625808	yes	yes	yes(41%)
FANCF-2	GGAACCCCGTCTGCAGCACCAGG	chr18	8707523	8707546	yes	yes	yes(12%)
FANCF-3	GGAGTCCCTCCTACAGCACCAGG	chr10	42914565	42914588	no	yes	no
FANCF-4	AGAGGCCCCTCTGCAGCACCAGG	chr17	80950160	80950183	yes	yes	no
FANCF-5	ACCATCCCTCCTGCAGCACCAGG	chrX	87100159	87100182	no	yes	no
FANCF-6	GGATTGCCATCCGCAGCACCTGG	chr6	143000000	143060965	no	yes	no(0.12%)
FANCF-7	TGAATCCCATCTCCAGCACCAGG	chr10	71703361	71703384	no	yes	no
FANCF-8	GGAGTCCCTCCTACAGCACCAGG	chr10	37664254	37664277	no	yes	no
FANCF-9	GGAGTCCCTCCTGCAGCACCTGA	chr16	49637107	49637130	no	yes	no

Table S11. PEAC-seq tag test

Tag No.	Tag sequence	Length (nt)	Nucleotide composition				Insertion efficiency on different sites			
			A%	C%	G%	T%	*EMX1*	*HEK3*	*RNF2*	*PRNP*
tag1	CACCACCATCACCACCAC	18	33.30%	61.10%	0.00%	5.60%	31.29%	86.19%	54.41%	81.65%
tag2	ATGATGGTGATGATGGTG	18	22.20%	0.00%	44.40%	33.30%	9.50%	51.11%	2.80%	26.57%
tag3	ATAACTTCGTATAAGAAAC CATATACGAAGTTAT	34	44.10%	29.40%	11.80%	14.70%		47.80%		
tag4	ATAACTTCGTATAGGGGGG GGTATACGAAGTTAT	34	29.40%	29.40%	32.40%	8.80%		41.19%		

Table S12. Optional PEAC-seq sequences

Tag No.	Tag sequence	Length (nt)	Nucleotide composition				Insertion efficiency at *HEK3* site
			A%	C%	G%	T%	
Optional PEAC-seq tag I	CATTACAACCATCACCACCATAAC	24	41.70%	16.70%	0.00%	41.70%	81.92%
Optional PEAC-seq tag II	CACTACAAACACTACTACCACAAG	24	45.80%	12.50%	4.20%	37.50%	77.60%
