# Supplementary material for: PEAC-seq adopts Prime Editor to detect CRISPR off-target and DNA translocation
Source: Nat Commun. 2022 Dec 12;13:7545. doi: 10.1038/s41467-022-35086-8 (PMC9744820; doi:10.1038/s41467-022-35086-8)
Supplement: Supplementary file 3 — Reporting Summary [file 41467_2022_35086_MOESM3_ESM.pdf]

nature portfolio

Corresponding author(s): Lijia Ma
Last updated by author(s): Nov 14, 2022

# Reporting Summary

Nature Portfolio wishes to improve the reproducibility of the work that we publish. This form provides structure for consistency and transparency in reporting. For further information on Nature Portfolio policies, see our Editorial Policies and the Editorial Policy Checklist.

## Statistics

For all statistical analyses, confirm that the following items are present in the figure legend, table legend, main text, or Methods section.

| n/a | Confirmed | |
|---|---|---|
| ☐ | ☒ | The exact sample size (*n*) for each experimental group/condition, given as a discrete number and unit of measurement |
| ☐ | ☒ | A statement on whether measurements were taken from distinct samples or whether the same sample was measured repeatedly |
| ☐ | ☒ | The statistical test(s) used AND whether they are one- or two-sided<br>*Only common tests should be described solely by name; describe more complex techniques in the Methods section.* |
| ☒ | ☐ | A description of all covariates tested |
| ☒ | ☐ | A description of any assumptions or corrections, such as tests of normality and adjustment for multiple comparisons |
| ☐ | ☒ | A full description of the statistical parameters including central tendency (e.g. means) or other basic estimates (e.g. regression coefficient) AND variation (e.g. standard deviation) or associated estimates of uncertainty (e.g. confidence intervals) |
| ☐ | ☒ | For null hypothesis testing, the test statistic (e.g. *F*, *t*, *r*) with confidence intervals, effect sizes, degrees of freedom and *P* value noted<br>*Give P values as exact values whenever suitable.* |
| ☒ | ☐ | For Bayesian analysis, information on the choice of priors and Markov chain Monte Carlo settings |
| ☒ | ☐ | For hierarchical and complex designs, identification of the appropriate level for tests and full reporting of outcomes |
| ☐ | ☒ | Estimates of effect sizes (e.g. Cohen's *d*, Pearson's *r*), indicating how they were calculated |

*Our web collection on statistics for biologists contains articles on many of the points above.*

## Software and code

Policy information about availability of computer code

| | |
|---|---|
| Data collection | SONY MA900 software (FACS data) |
| Data analysis | cutadapt version4.1, BWA version 0.7.13, SAMtools version 1.3, Integrative Genomics Viewer(IGV_2.8.13) , Graphpad prism8 |

For manuscripts utilizing custom algorithms or software that are central to the research but not yet described in published literature, software must be made available to editors and reviewers. We strongly encourage code deposition in a community repository (e.g. GitHub). See the Nature Portfolio guidelines for submitting code & software for further information.

## Data

Policy information about availability of data

All manuscripts must include a data availability statement. This statement should provide the following information, where applicable:

- Accession codes, unique identifiers, or web links for publicly available datasets
- A description of any restrictions on data availability
- For clinical datasets or third party data, please ensure that the statement adheres to our policy

GEO accessions: GSE179523, GSE179436
GRCh38 (GCA_000001405.15), GRCh37(GCA_000001405.1), mm10 (GCA_000001635.2), Epimap (https://epigenome.wustl.edu/epimap/data/imputed/)

## Human research participants

Policy information about studies involving human research participants and Sex and Gender in Research.

| | |
|---|---|
| Reporting on sex and gender | N/A |
| Population characteristics | N/A |
| Recruitment | N/A |
| Ethics oversight | N/A |

Note that full information on the approval of the study protocol must also be provided in the manuscript.

# Field-specific reporting

Please select the one below that is the best fit for your research. If you are not sure, read the appropriate sections before making your selection.

[x] Life sciences [ ] Behavioural & social sciences [ ] Ecological, evolutionary & environmental sciences

For a reference copy of the document with all sections, see nature.com/documents/nr-reporting-summary-flat.pdf

# Life sciences study design

All studies must disclose on these points even when the disclosure is negative.

| | |
|---|---|
| Sample size | For comparison, we choose 8 sites which were commonly used in the previous off-target detection methods( such as GUIDE-seq, Circle-seq, Discover-seq) to prove our design. |
| Data exclusions | No data were excluded |
| Replication | No replicates performed in this paper. We evaluated our results with comparison to the other similar job, such as GUIDE-seq, and performed further validation by amplicon-seq. |
| Randomization | We perform our study in cell line and animals with same background. Randomization is not relevant to the studies. |
| Blinding | Blinding was not relevant to our studies, because some primer sets was designed according to the protospacer, so we need to know the correspondence between the sample and the corresponding target sites. |

# Reporting for specific materials, systems and methods

We require information from authors about some types of materials, experimental systems and methods used in many studies. Here, indicate whether each material, system or method listed is relevant to your study. If you are not sure if a list item applies to your research, read the appropriate section before selecting a response.

## Materials & experimental systems

| n/a | Involved in the study | |
|---|---|---|
| x | | Antibodies |
| | x | Eukaryotic cell lines |
| x | | Palaeontology and archaeology |
| | x | Animals and other organisms |
| x | | Clinical data |
| x | | Dual use research of concern |

## Methods

| n/a | Involved in the study | |
|---|---|---|
| x | | ChIP-seq |
| | x | Flow cytometry |
| x | | MRI-based neuroimaging |

# Eukaryotic cell lines

Policy information about cell lines and Sex and Gender in Research

| | |
|---|---|
| Cell line source(s) | The Hek293T was purchased from ATCC. |
| Authentication | None of the cell lines used were authenticated. |
| Mycoplasma contamination | The cell lines were not tested for mycoplasma contamination. |
| Commonly misidentified lines (See ICLAC register) | There were no commonly misidentified lines used in this study. |

# Animals and other research organisms

Policy information about studies involving animals; ARRIVE guidelines recommended for reporting animal research, and Sex and Gender in Research

| | |
|---|---|
| Laboratory animals | 6-week-old C57BL/female mice;8-week-old C57BL/males mice;6-week-old ICR female mice;8-week-old ICR male mice; |
| Wild animals | No wild animals were used in the study. |
| Reporting on sex | Our study was performed with mouse embryos. So the sex was not considered in study design. |
| Field-collected samples | No field collected samples were used in the study. |
| Ethics oversight | Laboratory Animal Resource Center (LARC) at the Westlake University |

Note that full information on the approval of the study protocol must also be provided in the manuscript.

# Flow Cytometry

## Plots

Confirm that:

- [x] The axis labels state the marker and fluorochrome used (e.g. CD4-FITC).
- [x] The axis scales are clearly visible. Include numbers along axes only for bottom left plot of group (a 'group' is an analysis of identical markers).
- [x] All plots are contour plots with outliers or pseudocolor plots.
- [x] A numerical value for number of cells or percentage (with statistics) is provided.

## Methodology

| | |
|---|---|
| Sample preparation | HEK-293T cell line were collected with 0.05% trypsinization and filtration with 100 um strainer |
| Instrument | Sony MA900 |
| Software | Sony MA900 |
| Cell population abundance | 80-90% of total cells were live cells. 90-99% of live cells were single cells. 30-40% of single cells were GFP positive cells. 98% of post-sort cells were GFP positive. |
| Gating strategy | FSC-A and SSC-A for live cells; FSC-A and FSC-H for single cells; SSC-A and FITC-A for GFP positive cells. |

- [x] Tick this box to confirm that a figure exemplifying the gating strategy is provided in the Supplementary Information.
