# Supplementary material for: PEAC-seq adopts Prime Editor to detect CRISPR off-target and DNA translocation
Source: Nat Commun. 2022 Dec 12;13:7545. doi: 10.1038/s41467-022-35086-8 (PMC9744820; doi:10.1038/s41467-022-35086-8)
Supplement: Supplementary file 14 — Description of Additional Supplementary Files [file 41467_2022_35086_MOESM14_ESM.pdf]

Title: Supplementary Data 1
Description: In Cellulo(293T) *VEGFA TS1* off-target sites called by PEAC-Seq 6 set primers

Title: Supplementary Data 2
Description: In Cellulo(293T) *VEGFA TS2* off-target sites called by PEAC-Seq 6 set primers

Title: Supplementary Data 3
Description: In Cellulo(293T) *VEGFA TS3* off-target sites called by PEAC-Seq 6 set primers

Title: Supplementary Data 4
Description: In Cellulo(293T) *EMX1* off-target sites called by PEAC-Seq 6 set primers

Title: Supplementary Data 5
Description: In Cellulo(293T) *RNF2* off-target sites called by PEAC-Seq 6 set primers

Title: Supplementary Data 6
Description: In Cellulo(293T) *FANCF* off-target sites called by PEAC-Seq 6 set primers

Title: Supplementary Data 7
Description: In vivo (**C57BL/6**) *PCSK9* off-target sites called by PEACSeq 6 set primers

Title: Supplementary Data 8
Description: In vivo (**C57BL/6**) *Pnpla3* off-target sites called by PEACSeq 6 set primers

Title: Supplementary Data 9
Description: Primers, oligos and vectors used in the development of PEAC-seq technique

Title: Supplementary Data 10
Description: Primers and vectors used in in vivo IVT, PEAC-seq and Amplicon-NGS
